# Insights into polyethylene biodegradative fingerprint of *Pseudomonas citronellolis* E5 and *Rhodococcus erythropolis* D4 by phenotypic and genome-based comparative analyses

**DOI:** 10.3389/fbioe.2024.1472309

**Published:** 2024-12-12

**Authors:** Jessica Zampolli, Elena Collina, Marina Lasagni, Patrizia Di Gennaro

**Affiliations:** ^1^ Department of Biotechnology and Biosciences, University of Milano-Bicocca, Milan, Italy; ^2^ Department of Earth and Environmental Sciences, University of Milano-Bicocca, Milan, Italy

**Keywords:** polyethylene biodegradation, *Rhodococcus erythropolis*, *Pseudomonas citronellolis*, genome analysis, laccase activity, gene clusters

## Abstract

Polyethylene (PE) is the most-produced polyolefin, and consequently, it is the most widely found plastic waste worldwide. PE biodegradation is under study by applying different (micro)organisms in order to understand the biodegradative mechanism in the majority of microbes. This study aims to identify novel bacterial species with compelling metabolic potential and strategic genetic repertoires for PE biodegradation. *Pseudomonas citronellolis* E5 is newly isolated from solid organic waste contaminated with plastic debris, and *Rhodococcus erythropolis* D4 was selected for its promising potential in biodegradable plastic determined by its genetic repertoire. *P. citronellolis* E5 was selected for its ability to grow on PE as the only carbon and energy source. Meaningful extracellular secreted laccase activity was also characterized for D4 during growth on PE (E5 and D4 strains have a laccase activity of (2 ± 1)×10^–3^ U mg^−1^ and (3 ± 1)×10^–3^ U mg^−1^, respectively). Despite the highest level of cell numbers recorded at 7 days of growth on PE for both strains, the patterns of the metabolic products obtained and degraded during 60 days on PE were dissimilar in the two bacteria at different sampling times. However, they mainly produced metabolites belonging to carboxylic acids and alkanes with varying numbers of carbons in the aliphatic chains. Whole-genome sequence analyses of *P. citronellolis* E5 compared to *R*. *erythropolis* D4 and genetic determinant prediction (by gene annotation and multiple sequence alignment with reference gene products) have been performed, providing a list of 16 and 42 gene products putatively related to different metabolic steps of PE biodegradation. Altogether, these results support insights into PE biodegradation by bacteria of the *Pseudomonas* and *Rhodococcus* genera from metabolic and genetic perspectives as a base to build up novel biotechnological platforms.

## 1 Introduction

In the last decades, plastic exploitation has become a relevant issue due to its exceptional production rates and the consequent difficult disposal management at the end of useful life. Among the possible fates of plastic polymers, recycling and biodegradation processes involving microorganisms are the most eco-friendly methods (Lee and Liew, 2021). Indeed, a few microorganisms can break down polymers into simpler compounds by biochemical transformations through several steps, including biodeterioration, biofragmentation of the deteriorated plastics, generating plastic fragments with lower molecular weights or oxidized molecules, assimilation, and mineralization (Danso et al., 2019; Mohanan et al., 2020). Microbial assimilation routes are activated, forming biofilms on the plastic surface and/or producing active catalytic enzymes able to oxidize the polymers. The mineralization process comprises the transport of oxidized plastic derivatives into the cells, where diverse enzymatic reactions lead to complete degradation into oxidized metabolites, including CO_2_ and H_2_O (Zhang et al., 2022; Amobonye et al., 2021). Therefore, plastic polymer biodegradation using microorganisms equipped with the necessary enzymatic arsenals is a potential strategy for addressing waste plastics.

Synthetic plastics comprehend both hydrolyzable and non-hydrolyzable polymers. Polyethylene-based plastic is considered a non-hydrolysable polyolefin and the most recalcitrant to biodegradation, persisting in the environment for a long time. Polyethylene (PE) can be distinguished in high-density (HDPE) and low-density (LDPE) according to the molecular weight, 3-dimensional structures showing different degrees of branching, and availability of functional groups on the polymer surface (Sen and Raut, 2015; Montazer et al., 2020). PE is one of the most used materials in the manufacture of single-use plastic materials and, specifically, LDPE is mainly used to make carry bags and food packaging materials.

Since the early 2000s, PE biodegradation has been under study by applying bacterial and/or fungal pure cultures isolated from different environments, microbial consortia, and invertebrate mealworms (Bonhomme et al., 2003; Ray et al., 2023). The biodegradative process is accomplished by producing modifications in the functional groups, generating oxidized oligomers or products, and altering tensile properties and molecular weight, among other properties (Soleimani et al., 2021).

One of the major bottlenecks of such a treatment strategy and in defining a chemical as biodegradable is the duration of degradation, which should be limited to 28 days as in the cases reported in the standardized biodegradation test of OECD and ISO (ready biodegradability tests, inherent biodegradability tests, and simulation tests) (Strotmann et al., 2023). For example, OECD 301 indicates that 60% of the initial concentration or calculated reference value for biochemical oxygen demand or CO_2_ production are considered exceedance values.

Increasing numbers of research studies are focused on isolating and identifying new potential microbial degrading strains for plastic reduction, elimination, or potential recycling. They have been isolated from various sources, including waste-contaminated soils, compost, mangrove sediments, waste of the mulch films, landfills, dumping sites, marine environments, river estuaries, remote environments (Antarctica), and sewage treatment plants (Miri et al., 2022; Thakur et al., 2023). The common goal is to evaluate the metabolic potential for polymer biodegradation, improve its oxidation and biodegradation, and comprehend in-depth the process mechanism. Polyolefins like PE showed the highest recalcitrance and required an additional effort to identify the bacteria with the highest biodegradative potential. The main bacterial genera involved in PE biodegradation are *Bacillus*, *Pseudomonas*, and *Rhodococcus* (Soleimani et al., 2021; Montazer et al., 2020). However, not all bacteria species can adhere to hydrophobic surfaces, often requiring polymer pretreatment to obtain hydrophobic groups on their surfaces (Soleimani et al., 2021). Bacterial members of the last two genera are noteworthy in the field of biodegradation of organic matter, xenobiotic compounds, various contaminants, and plastic polymers (Zampolli et al., 2019; Zampolli et al., 2022; Medić and Karadžić, 2022; Shilpa et al., 2023; Kim et al., 2024).

A few enzymatic functions have been associated with the PE oxidative process such as laccase-like multicopper oxidases (EC 1.10.3.2.), manganese peroxidases (EC 1.11.1.13), and lignin peroxidases (EC 1.11.1.14) (Wei and Zimmermann, 2017; Andler et al., 2022; Zampolli et al., 2023; Ray et al., 2023). The study of the PE metabolic pathway in *Rhodococcus* bacteria indicated a plausible scenario of genes involved in the degradation of either UV-treated or untreated PE (Santo et al., 2013; Gravouil et al., 2017; Zampolli et al., 2021; Tao et al., 2023; Mishra et al., 2024; Kim et al., 2024). Primarily, the activation of extracellular secreted genes encoding laccase-like multicopper oxidases, lipases, esterases, alcohol dehydrogenases, long-chain fatty-acid-CoA ligases, and lipid transporters in the presence of PE leads to the formation of PE oligomers, oxygenated products, or lower molecular weight isolate fragments. The subsequent involvement of membrane transporters and oxygenases, such as alkane monooxygenases/hydroxylases and cytochrome P450 hydroxylases, supports the cell entrance and the further intracellular PE oxidation, respectively (Gravouil et al., 2017; Zampolli et al., 2021; Tao et al., 2023; Mishra et al., 2024; Rong et al., 2024; Kim et al., 2024). Furthermore, genes encoding for enzymes involved in the central metabolic pathways were also upregulated, including genes of steps of β-oxidation (Tao et al., 2023; Mishra et al., 2024).

In this context, the aim of this study is the characterization of the PE biodegradative potential of two bacteria, *Pseudomonas citronellolis* E5 and *Rhodococcus erythropolis* D4, providing both metabolic characterization and identification of plausible genomic and genetic traits to aid the bioremediation of polyolefins in an eco-friendly process.

*P. citronellolis* E5 is a novel isolate from a solid organic waste contaminated with plastics from a waste treatment plant, and *R*. *erythropolis* D4 was previously selected for its promising potential in biodegrade plastics (Zampolli et al., 2022; Zampolli et al., 2024). The *R*. *erythropolis* D4 genome has been completely sequenced and revealed to carry genes involved in plastic removal (Zampolli et al., 2022). These two bacteria were assayed for their ability to grow on PE as the sole carbon and energy source, evaluating their laccase enzymatic activity in the extracellular environment after growth on PE and the profile of metabolic products from PE oxidation solvent-extracted and analyzed by gas chromatographic analysis coupled with mass spectrometry. Their genome analysis (whole-genome alignment, annotation search, and gene clustering against reference sequences) predicted gene products related to PE biodegradation, hinting at their genetic potential for PE mineralization.

## 2 Materials and methods

### 2.1 Bacterial strain isolation and growth conditions

The bacterial isolation process was conducted using solid waste with plastic debris and microplastics (Supplementary Figure S1) sampled from the final treatment step biowaste treatment plant in Lombardy, Italy, with the same procedure described by Zampolli et al. (2023) after shredding the waste into small particles (1 cm–100 μm). At the end of the enrichment culture process in the presence of the solid waste, the individual bacterial colonies were isolated on Luria–Bertani (LB) agar plates and screened for their capacity to grow on solid M9 mineral medium (Maniatis et al., 1982) supplemented with 1% *v/v* Tween80 (as dispersing agent) with added 1% *w/v* sterile commercial low-density (untreated) powder PE (M9-Tw-PE) (Merck Darmstadt, Germany, Mn = 1700 by GPC, Mw = 4000 by GPC, cod. 427772). The sterility of the PE powder was obtained by UV radiation for 1 h using a 15 W UV-C germicidal lamp (Gelaire Flow Laboratories, Italy). The plates were then incubated at 30°C for 4 days to check for growth of colonies. This pre-screening was repeated three times. The novel isolated bacteria were first morphologically characterized by growth on the LB agar medium and M9-PE agar plates. The Gram stain was examined under a microscope with a magnification of up to ×100 (Axiolab E re, Carl Zeiss, Germany). This preliminary screening was also applied to another plastic-degrading bacterial strain, *R. erythropolis* D4, selected for its plastic metabolic potential demonstrated in previous works based on genetic trait identification (Zampolli et al., 2022; Zampolli et al., 2023). *R. erythropolis* (identification number 8B-C2-LD D4) (Zampolli et al., 2023) and a selected bacterial strain among the seven isolates from a plastic and organic waste treatment plant, *P. citronellolis* (identification number A-C1-1 E5), are deposited in the Private Collection of Microbiology Laboratory - BtBs Department of University of Milano-Bicocca.

All the new isolates and *R. erythropolis* D4 were periodically maintained in the M9-PE at 30°C. After washing their cells in the M9 mineral medium twice, they were pre-cultured in the M9 mineral medium supplemented with 20 mM malate (M9-M) and incubated at 30°C overnight.

The bacterial cells deriving from pre-cultures in M9-M were washed and resuspended in a fresh M9, then inoculated in 20 mL flasks in the presence of 1% *w/v* sterile low-density powder PE (M9-PE) as the sole carbon and energy source. Each bacterial culture was inoculated from the corresponding pre-culture to obtain an initial optical density read at 600 nm (OD_600_) of 0.1. All cultures were incubated at 30°C under shaking (120 rpm) for up to 7 days and sampled at 0 days, 1 day, 2 days, 3 days, 4 days, and 7 days to monitor their growth by measuring OD_600_.

### 2.2 Enzymatic activity

The bacterial isolates were discriminated by their enzymatic activity by evaluating both the intracellular and extracellular laccase activity because the isolates could activate laccase-like enzymes to perform the first oxidation of PE. After the bacterial cells were grown on 100 mL M9-PE for 3 days or 20 mL M9-M for 24 h, they were harvested by centrifugation (8,000 rpm), resuspended in 50 mM potassium phosphate buffer (pH 7, PPB), vortexed for 10 min, and incubated with 50 mg mL^−1^ lysozyme at 30°C for 1 h. Then, the cells were lysed by sonication (10 cycles of 20 s with 20% amplitude) with a Digital Sonifier™ (BRANSON Ultrasonic Corporation, Italy), and the soluble protein fraction was obtained by centrifugation at 4,000 rpm for 10 min at 4°C.

In the case of M9-PE cultures, the separated supernatant was filtrated to remove the residual PE using 0.45 μm filters (Millipore, Italy). The obtained cell-free supernatant fraction (CFS) was lyophilized (LIO5P Digital, 5Pascal, Italy) to maintain the enzymatic activity and resuspended in 50 mM PPB in a twenty-fifth of the initial volume. *R. erythropolis* D4 culture was also assessed for laccase activity.

Laccase enzymatic activity was determined as described by Zampolli et al. (2021) at 470 nm (ε: 36 mM^−1^ cm^−1^), also by adding 100 μM CuSO_4_. The presence of CuSO_4_ could enhance laccase activity, as observed by Santo et al. (2013) and Zampolli et al. (2023). One unit of enzyme activity is defined as the amount of enzyme that oxidizes 1 μmol per minute under the assay conditions. The specific activity was calculated as U mg^−1^ and reported as the mean of three replicates ± standard deviation (SD).

The total protein concentrations of the lyophilized CFS and cell extracts were assessed using the method of Bradford (1976) using Coomassie brilliant blue with bovine serum albumin as a standard. Protein concentrations were calculated from the standard curve by 20 μg mL^−1^ bovine albumin serum. All the experiments were performed in triplicate and shown as mean ± SD.

### 2.3 Kinetics of growth on PE

A biodegradative assay was performed to evaluate the ability to grow and degrade UV-sterilized untreated PE of the novel selected isolates, *P. citronellolis* strain E5 and *R. erythropolis* strain D4. Each bacterial culture was established in 50 mL flasks using liquid M9-PE (powder), and they were sampled at different times within a 60-day period (0 days, 3 days, 7 days, 14 days, 28 days, and 60 days) to evaluate the optical density and the total amount of bacterial cells using the colony-forming unit (CFU) count method by serially diluting 0.1 mL of each bacterial culture in a solution of M9 medium and plating the diluted suspension on LB agar medium. The plates were incubated at 30°C for 48 h. Both OD_600_ and CFU ml^−1^ data are reported as the means of three biological replicates with SD.

*P. citronellolis* E5 and *R. erythropolis* D4 were preliminarily tested for their ability to grow on metabolic intermediates of PE degradation, such as alkanes, ketones, and carboxylic acids. The growth assays were established in 20 mL flasks with 1 g/L *n*-dodecane, *n*-hexadecane, 2-hexadecanone, hexanoic acid, or dodecanoic acid to measure the optical density (each culture was established from OD_600_ ∼ 0.1) up to 72 h.

### 2.4 Biodegradation of polyethylene by GC-MS analyses

PE biodegradation was evaluated on the 20 mL M9-PE flask whole cultures, as reported in Section 2.1. M9-PE flasks without inoculum were used as abiotic controls. The biodegradation was monitored for up to 60 days (0 days, 3 days, 14 days, 28 days, and 60 days) by extracting the metabolic products from bacterial cultures with half a volume of dichloromethane (DCM) by manually shaking the separation funnel for 10 min and repeating the extraction twice. The samples were derivatized as reported in Zampolli et al. (2024) before injecting 1 µL in a 6890 N Network gas chromatograph (GC) system (J&W DB-5ms 60 m × 0.25 mm, 0.25 μm Ultra Inert GC Column, Agilent Technologies, Santa Clara, CA, United States of America) with helium at 99.99% as a carrier gas, coupled to a 5973 Network Mass Selective Detector (MSD, Agilent Technologies) at 70 eV in the scan ion monitoring mode (40–600 Da). The instrument was set as described in Zampolli et al. (2023). MSD ChemStation E.02.02.1431 (Agilent Technologies, Santa Clara, CA, United States of America) was used to analyze the resulting chromatograms. Unknown products were identified by comparing their mass spectra with the NIST11 database as long as their similarity with respect to the reference mass spectra was equal to or greater than 90%. The products obtained in each condition were classified according to different chemical classes, their peak areas were area normalized, and the proportion of different product types was calculated according to the “compound peak area/total compounds peak area × 100” (Zampolli et al., 2023). All the experiments were carried out in triplicates.

### 2.5 Nucleic acid extraction and manipulation

The total DNA was extracted from bacterial cells grown on M9-PE by the DNeasy UltraClean Microbial Kit (Qiagen, Italy) following the manufacturer’s instructions.

Long Range DNA Rabbit Polymerase (2.5 U μl^−1^, Eppendorf, Germany) was used for 16S rRNA gene amplification using the universal bacterial primers 27 F (AGA​GTT​TGA​TCC​TGG​CTC​AG) and 1495 R (CTA​CGG​CTA​CCT​TGT​TAC​GA) (Lane, 1991) (melting temperature, Tm 55°C) with the following thermocycling conditions: at 95°C for 2 min, 95°C for 30 s, specific Tm for 30 s, 72°C for 1 min and 45 s, for 30 cycles; and 72°C for 7 min. One-fifth of the PCR product was verified on 0.8% agarose gel using a 500–10,000 bp molecular ladder (Merck, Italy). Then, the PCR product was purified from the agarose gel with the kit NucleoSpin Gel and PCR Clean-up (Macherey–Nagel, Dueren, Germany), and the purified product was sequenced by the Sanger method. The nucleotide sequence of the 16S rRNA gene was obtained by comparing forward and reverse sequences, and the final gene fragment was subsequently compared to the NCBI database with BLASTn (Altschul et al., 1990) and to the SILVA database (Quast et al., 2013).

### 2.6 Genome sequencing and preliminary analyses

The total DNA of *P. citronellolis* E5 was sequenced by Illumina MiSeq v3 (2 × 300 bp; Illumina Inc., San Diego, CA). The library was obtained using Nextera XT DNA Library Preparation Kit with the standard Illumina DNA shotgun library protocol (Illumina Inc., San Diego, CA). The total reads of the *P. citronellolis* E5 genome resulted in 1.48 million (total base pairs around 414.4 Mbp). Trimmomatic v.0.38 (Bolger et al., 2014) was applied for the trimming process to discard the sequences with a per base sequence quality score of less than 30. The quality of the genome was evaluated using FastQC v0.11.7 (Andrews, 2010) before and after the trimming process. The resulting sequences were then assembled into contigs using Spades 3.15.5 (Bankevich et al., 2012). MeDuSa v1.6 enabled refining and assembling the contigs into scaffolds (Bosi et al., 2015), using the complete genomes of the following *Pseudomonas* strains as references: *P*. *citronellolis* WXP-4 (NCBI BioProject PRJNA511406), *Pseudomonas putida* KT2440 (PRJNA836759), and *Pseudomonas hydrolytica* DSWY01 (PRJNA509263).

Genome assembly quality (completeness) was determined considering the total assembly length (expected to be ∼ 7 Mb), and contiguity metrics were assessed at the contig level using QUAST 5.0.1 (Gurevich et al., 2013).

The genome was automatically annotated using the Rapid Annotation using Subsystem Technology (RAST) server (Aziz et al., 2008), Prokka (v. 1.14.6) (Seemann, 2014), and Bakta (v. 1.8.2) (Schwengers et al., 2021). The Comprehensive Antibiotic Resistance Database (CARD) and Resistance Gene Identifier (RGI; v. 6.0.3), CRISPR/Cas Finder (v. 4.2.20), and Phigaro (v. 2.3.0) were utilized to predict genes for antibiotic resistance (Alcock et al., 2020), for clustered regularly interspaced short palindromic repeats (CRISPR) (Couvin et al., 2018), and to detect and annotate prophage regions (Starikova et al., 2020). The main genomic features of *P*. *citronellolis* E5 and its comparison with *R. erythropolis* D4 genome were visualized by the Proksee viewer (https://proksee.ca/) (Accessed on 5 July 2024) (Grant et al., 2023).

EggNOG automatic classification was applied to evaluate the clusters of orthologous groups (COGs) for *P*. *citronellolis* E5 coding sequences (CDSs).

The genome sequencing data have been deposited in the European Nucleotide Archive (ENA) at EMBL-EBI under accession number PRJEB76139.

### 2.7 Phylogenetic analyses

In order to evaluate the phylogenesis of *P*. *citronellolis* E5 and *R*. *erythropolis* D4, a multi-locus sequence analysis (MLSA) was carried out by the Clustal W package of MEGA-X (version 10.2) using the default parameters (Kumar et al., 2018). MLSA was used to build the concatenated sequences, then inferred for tree development using the maximum likelihood (ML) method with a gamma distribution of mutation rates with gamma optimized to 2, 100 bootstrap replicates, and the Nearest-Neighbor-Interchange algorithm. The 16S rRNA, *rpoD*, and *ychF* marker genes were selected based on annotation and similarity; the *gyrB* gene for the *Pseudomonas* genus tree and the *secY* gene for the *Rhodococcus* genus tree were also selected. They were previously identified as conserved and informative for other bacterial classifications (Adékambi et al., 2011; Orro et al., 2015).

The phylogenetic tree of 14 *Pseudomonas* species (including *P. citronellolis* E5) was built using a dataset containing the following bacterial species: *P*. *citronellolis* (8), *Pseudomonas knackmussii* (2), *P*. *putida* (1), *Pseudomonas lalkuanensis* (1) *P*. *hydrolytica* (1), and one not-categorized species. The phylogenesis of 15 *Rhodococcus* species (including *R. erythropolis* D4) was developed with the following species: *R*. *erythropolis* (6), *Rhodococcus qingshengii* (1), *Rhodococcus ruber* (2), *Rhodococcus opacus* (3), *Rhodococcus jostii* (1), and two not-specified species. The strain species were selected based on the genome sequence availability.

### 2.8 Genomic comparison and genetic trait prediction in *P. citronellolis* E5 and *R. erythropolis* D4

Sequence and functional comparisons were performed between *P. citronellolis* E5 and *R*. *erythropolis* D4 utilizing a sequence-based and functional comparison tool on the RAST server. Average nucleotide identity (ANI) was also calculated by FastANI (v. 1.3.3) and the ANI calculator (Jain et al., 2018; Yoon et al., 2017; Liang et al., 2021). Enzyme functions and metabolic pathways were predicted by the Kyoto Encyclopedia of Genes and Genomes (KEGG) database by the KEGG Automatic Annotation Server (KAAS), providing functional annotation of genes by BLAST (Kanehisa et al., 2023).

The prediction of gene products potentially involved in PE biodegradation for *P. citronellolis* E5 and *R. erythropolis* D4 was conducted with two converging approaches. The first required the search into their genomes by annotation based on literature knowledge about PE metabolism and gene products retrieved from NCBI. The main enzymatic classes obtained by this approach were multicopper oxidases, peroxidase, lipases, esterases, alkane monooxygenases, and cytochrome P450 hydroxylases. A second method was amino acid (aa) sequence alignments carried out by Clustal Omega relying on annotation identifications and the selection of reference aa sequences (RASs) (Supplementary Table S1); 35 RASs were chosen to represent key steps of the PE biodegradative pathway, and they were clustered in a phylogenetic tree (Supplementary Figure S2). They were used in pairwise comparison with the retrieved aa sequences from the annotation search (first approach), selecting the gene products with the highest identity for comparison against the E5 and D4 genomes (second approach). The resulting gene products were also evaluated for the presence of aa translocation signal peptides using SignalP (v. 6.0) (Teufel et al., 2022) to predict their potential ability to be secreted in the extracellular environment.

The gene products obtained from these analyses were aligned by the Clustal Omega program using the default parameters of the multiple sequence alignment (MSA) tool (Sievers et al., 2011) with the same setting reported by Zampolli et al. (2024). In turn, a cluster tree was built using the selected RASs for each strain (E5 and D4). MSA served as input in MEGA software (version 10.2) by applying the maximum likelihood (ML) method (Kumar et al., 2018), with a JTT substitution matrix and a gamma distribution of mutation rates with gamma optimized to 2, inferring a phylogeny test with 50 bootstrap replicates.

## 3 Results

### 3.1 Isolation of bacterial strains from plastic-contaminated organic waste

A solid organic waste rich in plastic particles was obtained from an organic waste treatment plant. It exhibited a total microbial viable count of heterotrophic bacteria of around 2 × 10^6^ CFU mL^−1^. The isolation of novel plastic-degrading bacterial strains was performed by 7-day growth enrichment cultures supplemented with plastic-rich waste (Supplementary Figure S1). Thirty single bacterial colonies isolated from enrichment cultures and plated on LB agar medium were preliminarily screened for their ability to grow on PE agar plates (M9-Tw-PE). Seven isolated bacterial strains were able to grow on M9-Tw-PE plates for three subsequent repetitions. The isolates, named A, B, C, E5, F, H, and I, were characterized by a Gram stain, which revealed that A and E5 belonged to the Gram-negative group, and the remaining isolates belonged to the Gram-positive group.

**FIGURE 1:**
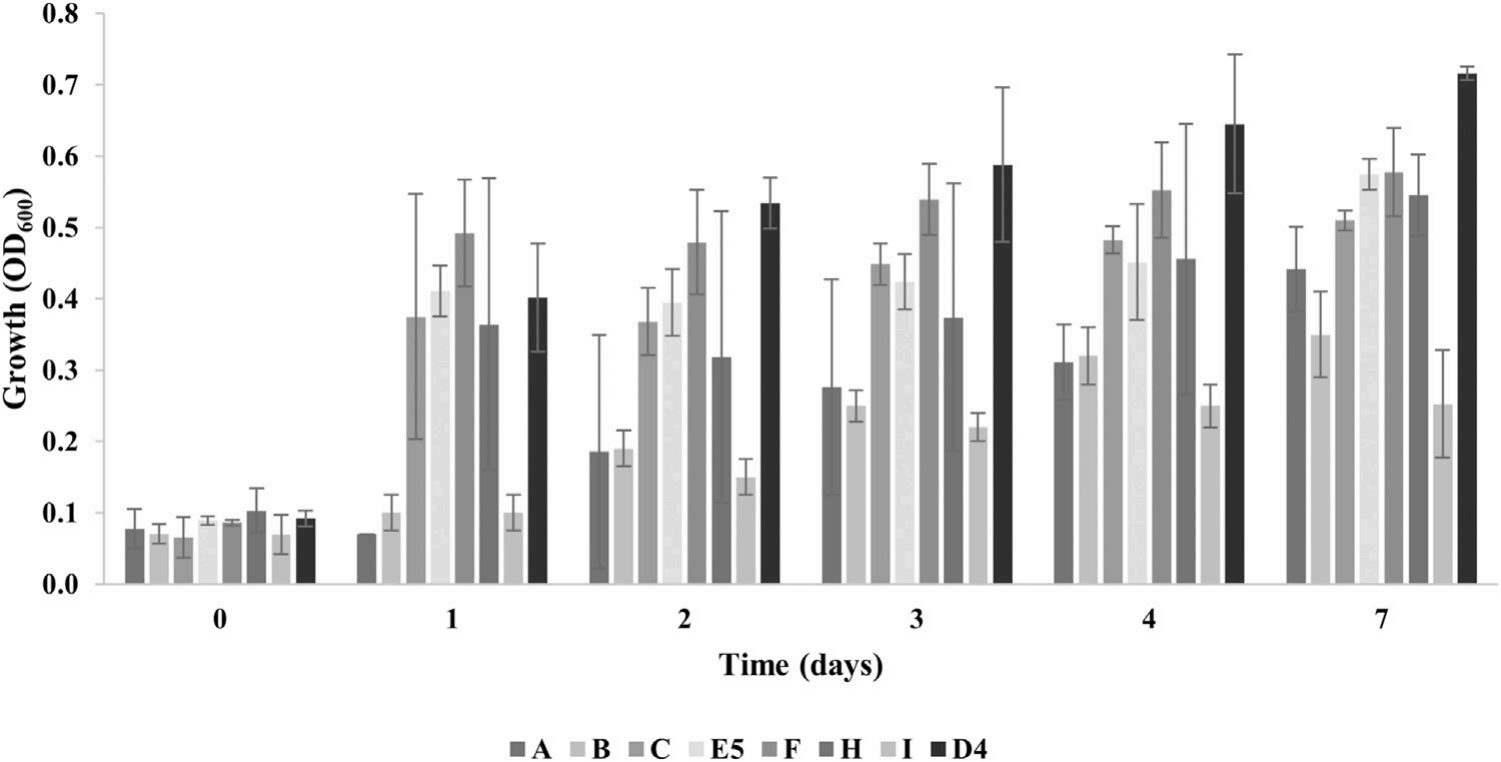
Growth levels of individual bacterial isolates (A, B, C, E5, F, H, and I) from plastic-contaminated organic waste compared to *R. erythropolis* D4 on 1% PE. The growth is presented as the mean value of the absorbance recorded at OD_600_ ± SD.

Then, the isolates were tested for their ability to grow in the presence of 1% PE as the sole carbon and energy source (M9-PE). In addition, the PE degradative ability of *R. erythropolis* D4 was also assessed because of its intriguing potential for plastic biodegradation, evidenced in previous studies (Zampolli et al., 2022; Zampolli et al., 2023). Figure 1 shows their growth assay measuring the OD_600_ for up to 7 days. At the final time of growth, the maximum OD_600_ level was ∼ 0.6, recorded for E5 (0.57 ± 0.01) and F (0.58 ± 0.02) isolates, compared to D4 growth (0.72 ± 0.08). Instead, the minimum level of growth was registered for the B (0.35 ± 0.06) and I (0.25 ± 0.09) isolates. Therefore, the isolates A, C, E5, F, and H were selected for subsequent investigations.

### 3.2 Screening of laccase activity during growth of plastic-degrading strains on untreated polyethylene

An enzymatic assay was used to evaluate the ability of the five selected isolated bacterial strains to secrete or produce intracellular oxidase enzymes for PE oxidation after 3 days of growth on PE, according to the hypothesis that they could activate laccase-like enzymes to perform the first oxidation of PE. The laccase activity was also assessed after the growth of the selected strains in M9-M as a reference condition. Both intracellular and CFS measurements showed a laccase activity of around one order of magnitude less than the respective conditions in M9-PE. The enzymatic assay was performed using 2,6-dimethoxyphenol (2,6-DMP) as one of the typical substrates for laccase activity both in the presence and in the absence of CuSO_4_ because copper is known to be a good enhancer of laccase activity (Santo et al., 2013; Zampolli et al., 2023).

**FIGURE 2:**
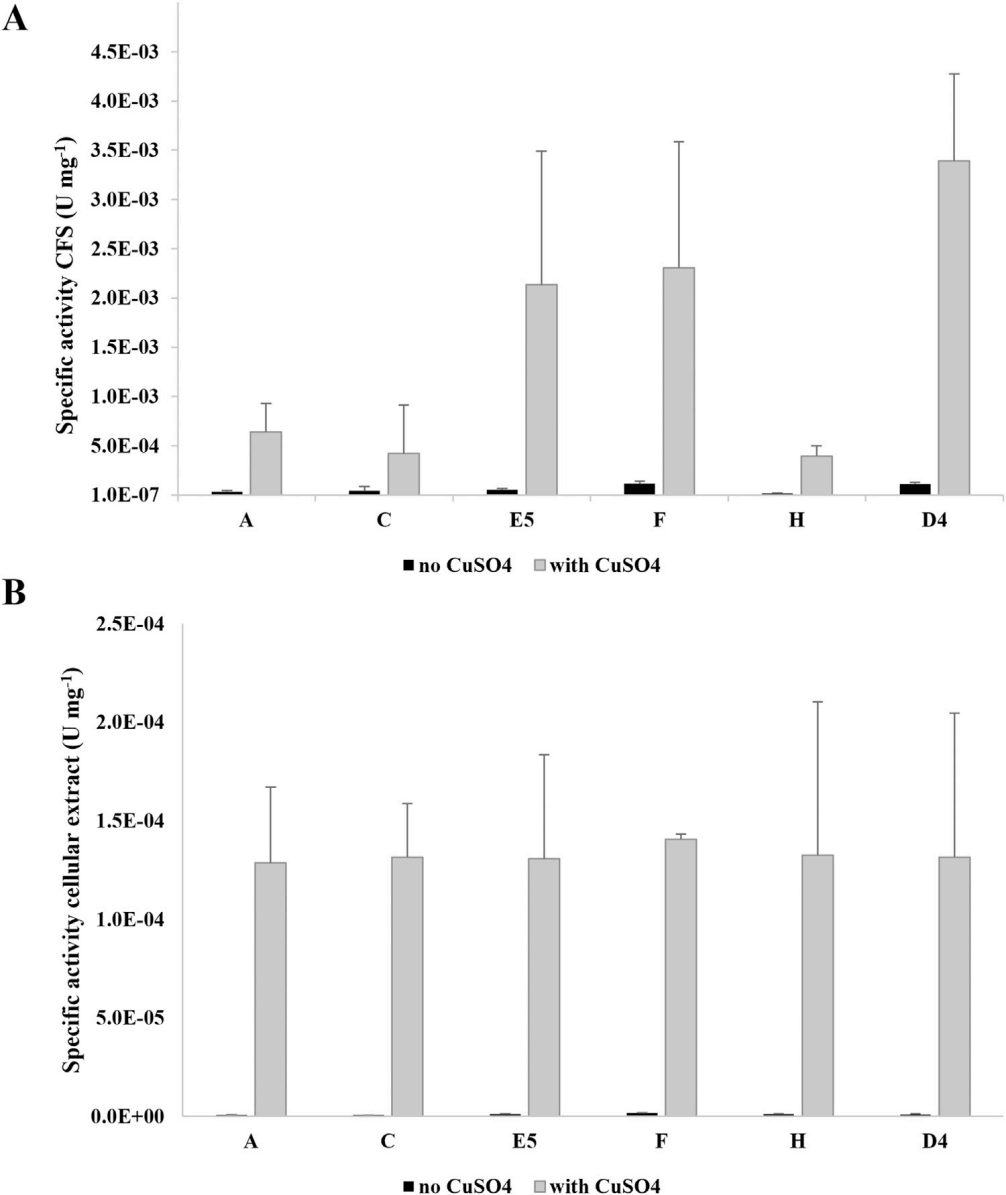
Specific laccase activity (U mg^−1^ of total proteins) of the supernatants (CFS) **(A)** and cellular extracts **(B)** of new isolates A, C, E5, F, and H and *R. erythropolis* D4. Laccase activity was measured in the presence of 2,6-DMP after growth on M9-PE in the absence (light gray) and in the presence of 100 µM CuSO_4_ (gray) added during the enzymatic assay. The laccase activity is the mean of three replicates ± SD.

Figures 2A, B show the laccase activity respectively secreted and intracellularly produced by the selected isolates A, C, E5, F, and H and *R. erythropolis* D4. For both cellular compartments, the highest activity was recorded in the presence of CuSO_4_ with a difference of at least one order of magnitude (CFS) or two to three orders of magnitude (cellular extracts) with respect to the condition of absence of CuSO_4_. Considering the condition with copper, the highest laccase activity was recorded in the CFS for all the tested strains. Among the bacterial isolates, the highest values were measured in CFS for E5 ((2±1) *10^–3^ U mg^–1^), F (2.3±0.1) *10^–3^ U mg-1), and R. erythropolis D4 ((3±1) *10^–3^ U mg^–1^) compared to intracellular values of around 10^–4^ U mg^−1^.

These results indicated that all the isolates were able to secrete active laccase-like enzymes during growth on PE.

### 3.3 Molecular identification of five isolates and phylogenetic analysis of *Pseudomonas citronellolis* E5 and *Rhodococcus erythropolis* D4

The five selected isolated bacteria were identified based on 16S rRNA gene amplification and sequencing. Silva analyses revealed that the 16S rRNA gene of the A, C, E5, F, and H isolate strains shared 99% identity with *Acinetobacter lwoffii*, 98% identity with *Priestia*/*Bacillus megaterium*, 98% identity with *P. citronellolis*, 98% identity with *Rhodococcus* sp., and 98% identity with *Bacillus* sp., respectively. Thus, considering the preliminary growth assays in M9-PE, the highest values of laccase activity were reported for *P*. *citronellolis* E5 and *Rhodococcus* sp. F. *P*. *citronellolis* strain E5 was selected for subsequent investigations of its PE biodegradative potential because its taxonomy is distant with respect to *R*. *erythropolis* D4, chosen for its promising genetic repertoire for polyolefin degradation (Supplementary Figure S3). The phylogenesis of these two selected strains was further evaluated by an MLSA and building a phylogenetic tree using the ML method considering, respectively, 13 *Pseudomonas* and 14 *Rhodococcus* species. The selected marker genes for the two different trees were identical (16S rRNA, *rpoD*, and *ychF*) except for the *gyrB* gene for the *Pseudomonas* genus and the *secY* gene for *Rhodococcus*.

**FIGURE 3:**
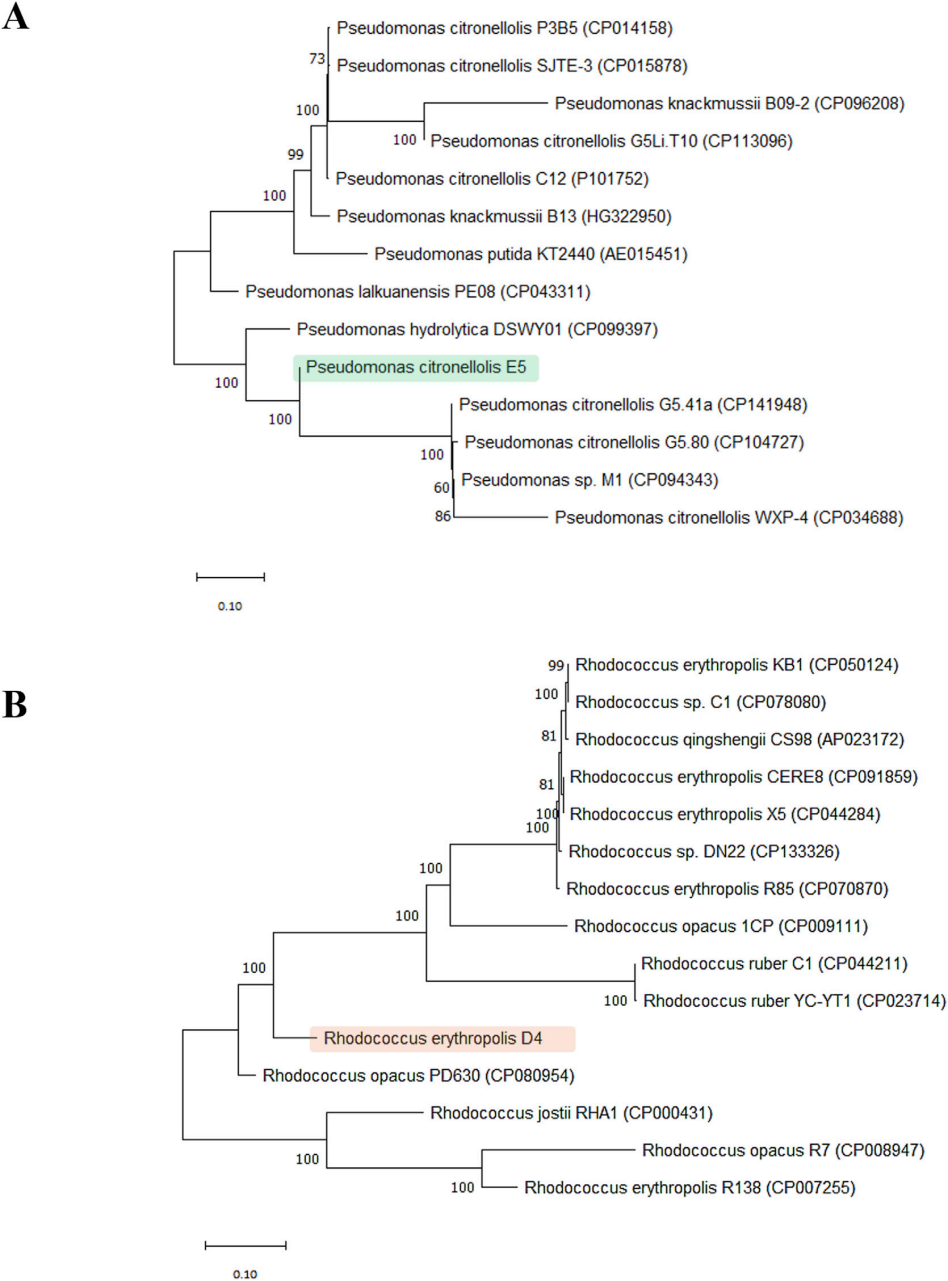
Phylogenetic trees. Phylogenetic analysis of *P. citronellolis* E5 **(A)** and *R. erythropolis* **(B)** based on sequence alignments with reference strains for each genus (13 *Pseudomonas* and 14 *Rhodococcus* species). The tree was constructed based on concatemer sequences of four marker genes: 16S rRNA, *rpoD*, *ychF*, and *gyrB* for the *Pseudomonas* genus and *secY* for the *Rhodococcus* genus.

Figure 3 shows the two distinct phylogenetic trees for *P. citronellolis* E5 (Panel A) and *R. erythropolis* D4 (Panel B). The clade of the E5 strain primarily includes *P. citronellolis* G5.41a and *P. citronellolis* G5.80. The taxonomical evaluation of *R. erythropolis* D4 validates that its closest neighborhood includes *R*. *jostii*, *R*. *opacus*, and other *R. erythropolis* strains.

### 3.4 Kinetic growth of *Pseudomonas citronellolis* E5 and *Rhodococcus erythropolis* D4 on untreated polyethylene

**FIGURE 4:**
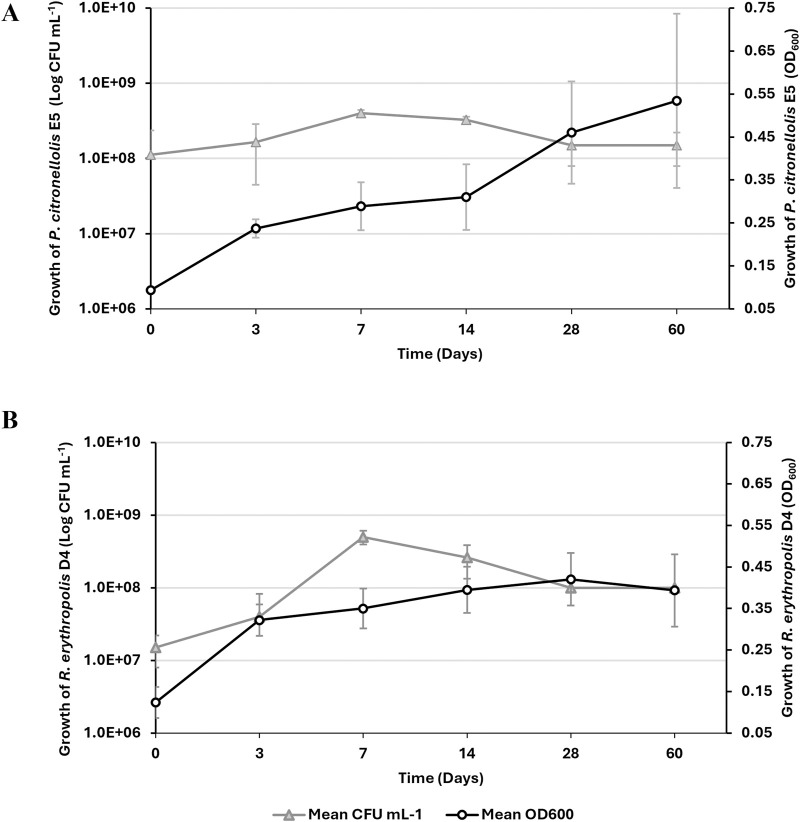
Growth kinetic assay of *P. citronellolis* E5 **(A)** and *R*. *erythropolis* D4 **(B)** on M9-PE up to 60 days. Growth is reported as the mean value of the absorbance recorded at OD_600_ (white circle) or counts of live bacterial cells expressed as CFU mL^−1^ (gray triangle) ± SD.

Based on the preliminary laccase screening after growth on PE, individual growth assays of the two selected strains in the presence of 1% PE as the sole carbon and energy source were performed for up to 60 days. Figure 4 shows the kinetic growth profile of *P. citronellolis* E5 (panel A) and *R. erythropolis* D4 (panel B) by optical density at OD_600_ and viable cell count. Considering the bacterial cell counts, the maximum level was reached at 7 days 4.0 ± 0.4 × 10^8^ CFU mL^−1^ and 5.0 ± 0.1 × 10^8^ CFU mL^−1^ for E5 and D4, respectively. However, only the D4 strain evidenced growth of around one order of magnitude in 7 days. For both strains, the density of cells underwent a slight decrease after the first week of growth. The maximum OD_600_ was reached at 60 days and 28 days for E5 (OD_600_ ∼ 0.53 ± 0.2) and D4 (OD_600_ ∼ 0.42 ± 0.06), respectively. These variations do not correspond to the value of total viable cell count because *R. erythropolis* D4 is prone to aggregate during growth on hydrophobic substrates (Zampolli et al., 2014). Because PE supports the growth of these selected bacteria, other evidence of PE catabolic degradation was investigated.

### 3.5 Biodegradation products of untreated polyethylene by GC-MS analyses

The biodegradation of PE for each M9-PE exhausted bacterial culture was recorded at 0 days, 3 days, 14 days, 28 days, and 60 days by evaluating the residual metabolic products extracted with DCM through GC-MS analyses. The resulting chromatographic profiles were compared to the ones obtained on untreated PE powder (UV-sterilized) that was not exposed to any (a)biotic degradation (no inoculum).

Unprocessed commercial PE powder (t0) was analyzed beforehand, evidencing mostly ketones with carbon chain lengths mainly ranging between C12 and C25, alkanes mainly ranging between C15 and C25, and carboxylic acids with C16 and C21 carbon chain lengths (Table 1).

**TABLE 1:** Metabolic products detected by GC-MS analyses deriving from untreated commercial PE biodegradation by *P. citronellolis* E5 and *R. erythropolis* D4.

Compound^a^	Retention time (t_R_) (min)	MW^b^	Formula	CAS number	t0	*Pseudomonas citronellolis* E5				*Rhodococcus erythropolis* D4				No inoculum		
						3 d^c^	14 d	28 d	60 d	3 d	14 d	28 d	60 d	14 d	28 d	60 d
Benzoic acid, TMS derivative^d^	13.5	194.3	C10H14O2Si	002078–12-8	-	-	-	●^e^	-	-	-	-	-	-	-	-
Octanoic acid, TMS derivative	13.7	216.4	C11H24O2Si	055494–06-9	-	-	-	-	-	●	-	-	-	-	-	-
2-Decanol, TMS derivative	13.9	230.5	C13H30OSi	053690–77-0	-	-	-	-	-	-	-	●	-	-	-	-
Decanoic acid, TBDMS derivative	14.5	286.5	C16H34O2Si	104255–74-5	-	-	-	●	●	●	●	-	-	-	-	-
2-Dodecanone	15.6	184.3	C12H24O	006175–49-1	●	-	-	-	-	-	-	-	-	-	●	●
Pentadecane	16.9	212.4	C15H32	000629–62-9	●	-	-	-	-	-	-	-	-	-	●	-
2-Tetradecanone	18.2	212.4	C14H28O	002345–27-9	●	-	-	-	-	-	-	-	-	●	●	●
Dodecanoic acid, TMS ester	18.7	272.5	C15H32O2Si	055520–95-1	-	-	-	●	-	-	-	-	●	-	-	-
Heptadecane	19.3	240.5	C17H36	000629–78-7	●	-	-	-	-	-	-	-	-	-	●	●
cis, 6-Octadecenoic acid, TMS ester/petroselinic acid, TMS derivative	19.6	354.6	C21H42O2Si	096851–53-5	-	●	-	-	●	-	-	-	-	-	-	-
5,5-Diethylpentadecane	20.43	268.5	C19H40	85977274 (CID)	-	-	●	●	●	●	-	-	-	-	-	-
2-Hexadecanone	20.46	240.4	C16H32O	002345–28-0	●	-	-	-	-	-	-	-	-	●	●	●
Tetradecanoic acid, TMS ester	20.9	300.5	C17H36O2Si	018603–17-3	-	-	-	●	●	-	●	●	●	-	●	●
n-Pentadecanoic acid, TMS ester	21.9	314.6	C18H38O2Si	074367–22-9	-	-	-	●	-	-	-	●	-	-	-	-
Methyl n-hexadecyl ketone	22.5	268.5	C18H36O	007373–13-9	●	-	-	-	-	-	-	-	-	●	●	●
Hexadecanoic acid, TMS ester	22.9	328.6	C19H40O2Si	055520–89-3	●	●	●	●	●	●	●	●	●	●	●	●
Dodecanedioic acid, bis(TMS) ester	23.3	374.6	C18H38O4Si2	022396–19-6	-	-	-	-	-	-	-	●	-	-	-	-
Nonadecane	23.46	268.5	C19H40	000629–92-5	●	-	-	-	-	●	-	-	-	-	●	●
10-Heptadecenoic acid, (Z)-, TMS derivative	23.6	340.6	C17H32O2	029743–97-3	-	●	●	●	●	-	-	-	-	-	-	-
2-Nonadecanone	24.45	282.5	C19H38O	000629–66-3	●	●	-	-	-	●	-	-	-	●	●	●
9,12-Octadecadienoic acid (Z,Z)-, TMS ester	24.46	352.6	C21H40O	056259–07-5	-	-	-	-	-	-	●	-	-	-	-	-
trans-9-Octadecenoic acid	24.5	282.4	C18H34O2	000112–79-8	-	-	-	-	-	-	●	-	-	-	-	-
Oleic acid, TMS trimethylsilyl ester	24.51	354.6	C21H42O2Si	021556–26-3	-	-	-	-	-	-	●	-	-	-	-	●
13-Octadecenoic acid, (Z)-, TMS derivative	24.55	354.6	C21H42O2Si	013126–39-1	-	●	-	-	-	-	-	-	-	-	-	-
Stearic acid, TMS derivative	24.7	356.6	C21H44O2Si	018748–91-9	●	-	●	●	●	-	●	●	●	●	●	●
Eicosane	25.2	282.5	C20H42	000112–95-8	-	-	-	●	-	●	●	●	●	-	●	●
10-Nonadecenoic acid, (Z)-, TMS derivative	25.4	368.6	C22H44O2Si	073033–09-7	-	-	●	●	-	-	-	-	-	-	-	-
Heneicosane	25.7	296.6	C21H44	000629–94-7	-	-	●	●	●	●	●	-	●	-	-	-
Tetracosane	26.1	338.6	C24H50	000646–31-1	-	-	●	-	●	-	●	●	●	-	-	●
2-Docosanone	26.2	324.6	C22H44O	077327–11-8	●	-	-	-	-	-	-	-	-	●	●	●
Eicosanoic acid, TMS	26.4	384.7	C23H48O2Si	055530–70-6	-	-	-	-	-	-	-	●	-	-	-	-
Pentacosane	26.9	352.6	C25H52	000629–99-2	●	-	●	-	-	-	●	●	-	-	-	●
Hexacosane	27.7	366.7	C26H54	000630–01-3	-	-	-	-	-	-	-	●	-	-	-	●
2-Pentacosanone	27.8	366.7	C25H50O	075207–54-4	●	-	●	-	-	●	-	●	-	●	●	●
Heptacosane	28.6	380.7	C27H56	000593–49-7	-	-	-	-	-	●	-	●	●	-	-	●
Octacosane	29.6	394.7	C28H58	000630–02-4	-	-	-	-	-	-	-	●	-	-	-	-
Nonacosane	30.7	408.7	C29H60	000630–03-5	-	-	-	-	-	-	-	●	-	-	-	-

^a^ Compound, the considered degradation products had more than 90% similarity with the mass spectra of NIST11.

^b^ MW, molecular weight of compounds by NIST11.

^c^ 3 d, number of days of incubation.

^d^ TMS, derivative, trimethylsilyl derivative.

●, the symbol “●” indicates the presence of the compound, while “-” indicates the absence.

**FIGURE 5:**
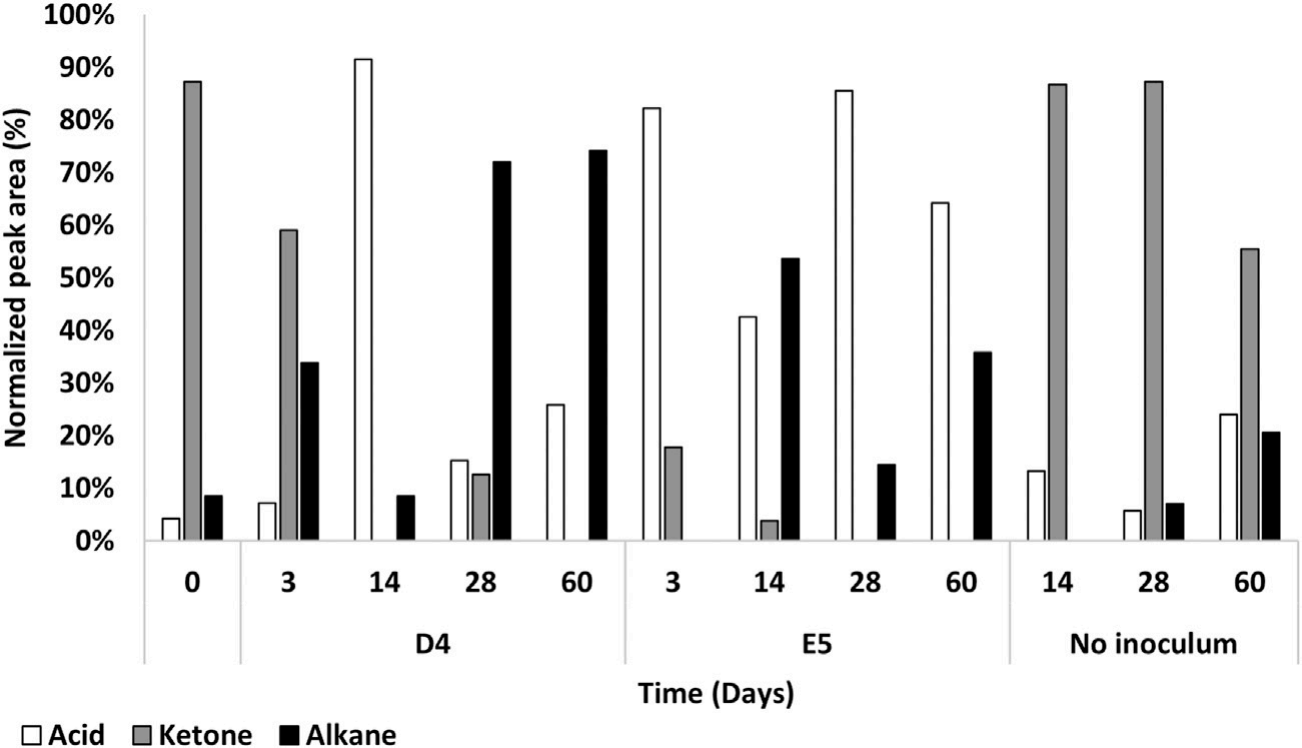
Percentage of normalized peak area of product types from *P. citronellolis* E5 and *R*. *erythropolis* D4 oxidation of PE with respect to the control (no inoculum) up to 60 days by GC-MSD analysis. The main product species after PE oxidation were carboxylic acids (white), ketones (gray), and alkanes (black).

The compounds released by the two strains at each time point are dissimilar as well as their production pattern (Table 1; Figure 5). After a 3-day incubation in M9-PE, an increase of carboxylic acids was detected. The main difference is the presence of a higher number of carboxylic acids for *P. citronellolis* strain E5, showing carboxylic acids with a carbon chain length of C16 and C18 and a monounsaturated fatty acid (petroselinic acid, trimethylsilyl (TMS) derivative, T_R_ = 19.6 min) (Table 1; Figure 5). In contrast, *R*. *erythropolis* D4 shows carboxylic acids with shorter carbon chain lengths (C8, C10, and C16). The number of ketone types decreased compared to the initial condition for both strains, while only *R*. *erythropolis* D4 produced alkane products ranging between C20 and C27 of carbon chain length. All these products indicated an initial oxidative process.

At 14 days, the trend of the number of product types and normalized peak area of fatty acids and alkanes inverted for the two strains; carboxylic acids increased, and alkanes decreased in the D4 culture, while the opposite was observed for the E5 culture.

After 28 days of cultivation, the number of product types and normalized peak area of fatty acids and alkanes inverted in the two strain cultures with respect to the previous sampling time. In addition, this pattern of metabolic products was stable at the successive time point (60 days) with a few differences in the types of oxygenated products (Figure 5). This indicates an oscillatory metabolism in which every oxidized product is subsequently degraded. After 1 month, *R*. *erythropolis* D4 shows an increase in aliphatic hydrocarbons with a higher number of carbon chain lengths (up to C29) with respect to *P. citronellolis* E5 (up to C21), and both strains show a diversity of carboxylic acids in addition to the hexadecanoic acid and octadecanoic acid that are always present. Interestingly, the presence of benzoic acid in the *P. citronellolis* E5 culture extract was indicative of a potential plastic antioxidant added to the PE released from its structure after 28 days (Zampolli et al., 2023; Yao et al., 2022; Sanluis-Verdes et al., 2022).

Finally, the not-inoculated condition showed the highest percentage of ketone types in all time points, as observed in the initial condition, and the variety of oxidized products is quite stable over time. This indicates no differences in terms of chromatographic profile between the initial time and the condition without inoculum (Supplementary Figure S4).

In order to support these findings, *P. citronellolis* E5 and *R. erythropolis* D4 were preliminarily tested for the ability to grow on the most representative metabolic intermediates of PE biodegradation, such as *n*-dodecane, *n*-hexadecane, 2-hexadecanone, hexanoic acid, or dodecanoic acid, as the sole carbon and energy source. Within 72 h (the first sampling time of strain grown on M9-PE), the highest growth level of D4 strain was on 2-hexadecanone, followed by *n*-hexadecane and hexanoic acid. Lower growth values were reported for *n*-dodecane and dodecanoic acid. For *P. citronellolis* E5, the highest growth levels were reported in the presence of hexanoic acid and dodecanoic acid. In contrast, in the presence of the tested alkanes and ketone, E5 growth levels were lower than the other substrates (Supplementary Figure S5).

### 3.6 Genome sequencing of *P. citronellolis* E5 and comparison with *R*. *erythropolis* D4 genome

The genome of *P. citronellolis* E5 was completely sequenced by Illumina MiSeq v3 (2 × 300 bp), obtaining 1.48 million reads (total base pairs around 414.4 Mbp). The sequencing quality showed that the shortest reads were ∼280 bp long, and the average genome coverage was 111X. After read quality trimming, the remaining reads with 70X coverage were assembled into 244 contigs with an N50 length of 313,394 bp. Contigs were assembled into 120 scaffolds by MeDuSa v1.6 using three reference genome sequences of bacteria of the *Pseudomonas* genus, resulting in a 7,075,332 bp genome size and an average G-C% of around 67%.

Automated annotation analysis of *P. citronellolis* E5 genome sequences was obtained using the RAST server, identifying a total of 6,527 open reading frames (ORFs), in agreement with the results obtained from the automated gene prediction and annotation by Prokka and Bakta software, which predicted 6,191 ORFs and 6,275 ORFs, respectively. The general genome analysis by RAST evidenced 72 RNA genes (12 rRNAs and 60 tRNAs) and 1,648 hypothetical proteins (HP).

**FIGURE 6:**
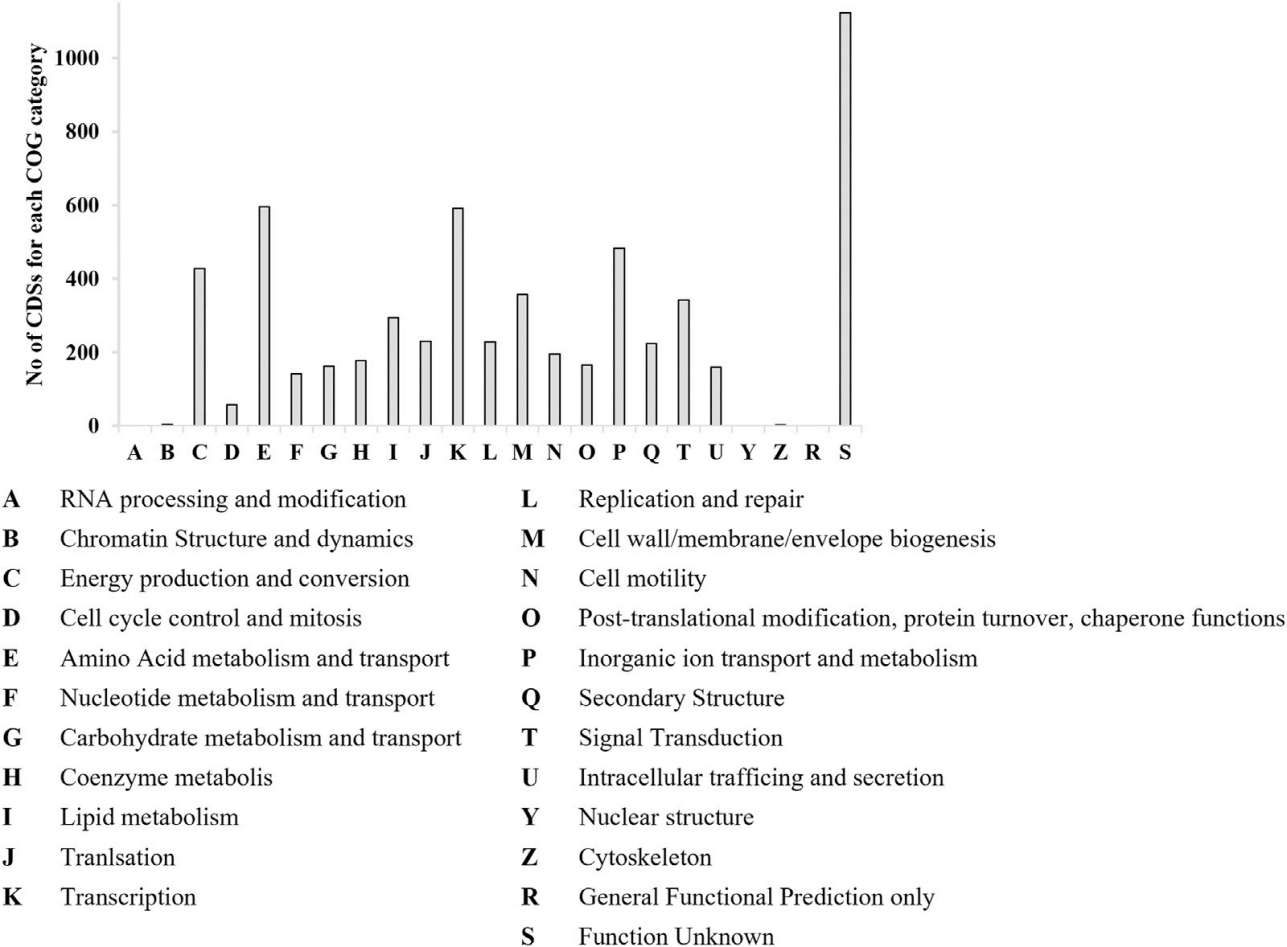
Number of CDSs populating each COG category by EggNOG categorization for *P. citronellolis* E5. The x-axis includes the abbreviations for each COG category.

*P. citronellolis* E5 CDSs were classified by EggNOG Automatic Classification based on clusters of COGs. The assigned COGs with the highest number of CDSs were amino acid metabolism and transport (E), 10%; transcription (K), 10%; inorganic ion transport and metabolism (P), 8%; energy production and conversion (C), 7%; unknown function (S), 16% (Figure 6).

**FIGURE 7:**
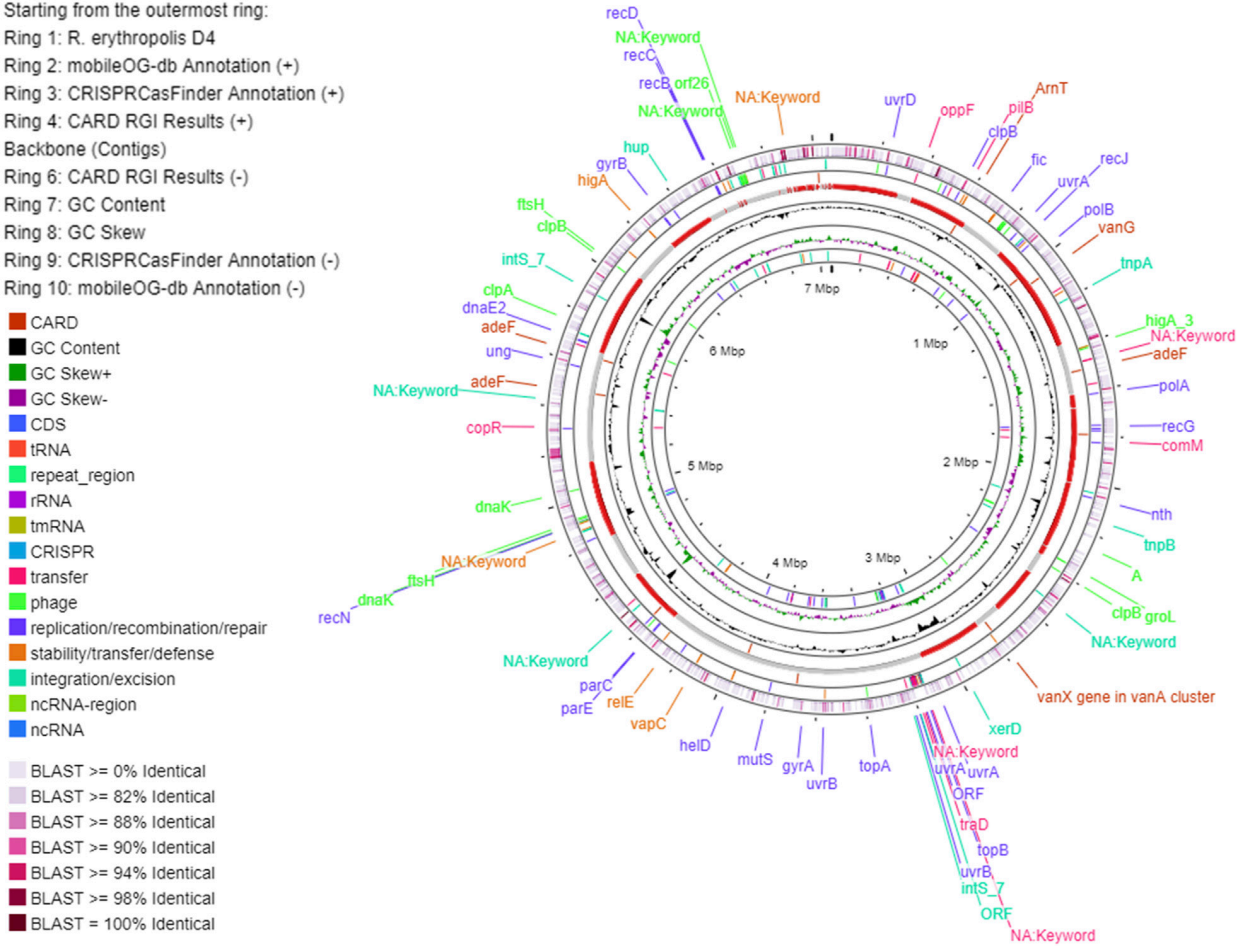
Circular map of *P. citronellolis* E5 genome predicted with Proksee viewer (https://proksee.ca/). The first outermost ring represents the BLAST comparison of the E5 genome with the genome of *R. erythropolis* D4 according to the sequence identity.

**FIGURE 8:**
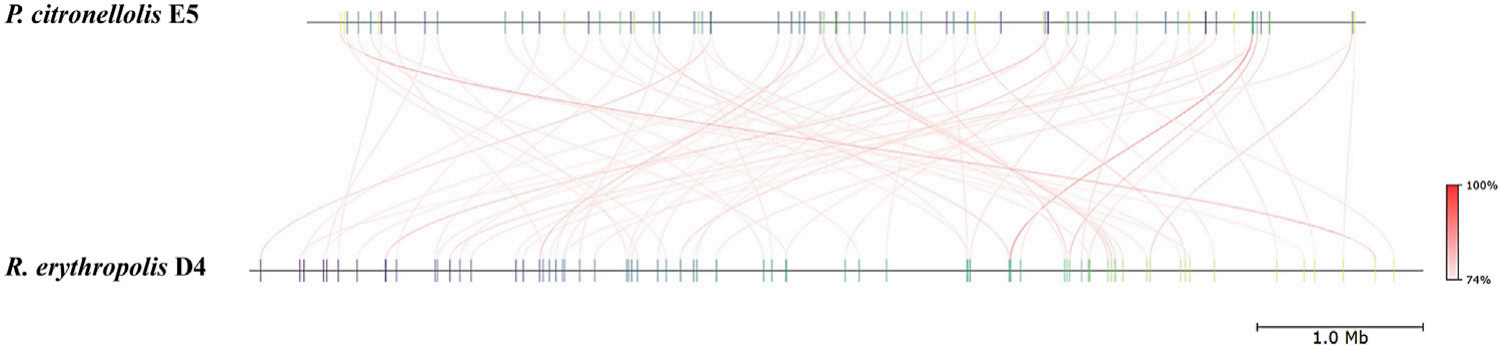
Pairwise comparison of *P. citronellolis* E5 and *R. erythropolis* D4 by whole-genome average nucleotide identity (ANI). Each strain genome is linearly depicted. The red lines indicate the correspondence between similar regions of the two genomes (95% threshold), thus representing the relatedness of the genome sequence. The heat map represents the ANI value for each orthologous match.

The main genome features of the E5 strain are depicted in Figure 7 showing G-C content, rRNA, tRNA, and tmRNA genes encoding for phages, replication or repair, transfer, stability/defense, integration, and excision, CRISPR-Cas, and antibiotic-resistant genes (ARG). In addition, putative horizontal gene transfer (HGT) events were predicted. In the same figure, the *R. erythropolis* D4 genome is depicted in the first outermost ring, indicating the BLAST comparison between the two genome sequences. This representation showed that a few CDSs have a higher identity percentage than 82%; thus, comparative analyses between the *P. citronellolis* E5 and *R. erythropolis* D4 genomes were carried out. The nucleotide-level genomic similarity (ANI) computed with different methods (Jain et al., 2018; Yoon et al., 2017; Liang et al., 2021) of E5 vs. D4 genomes ranges between 65% and 68% (Figure 8). The comparative analyses obtained using the RAST server indicated that the closest neighbors of *P. citronellolis* E5 genome are *Pseudomonas aeruginosa* PAO1 (score 547), *P. putida* KT2440 (score 524), *Pseudomonas syringae* pv. phaseolicola 1448A (score 523), and *Pseudomonas fluorescens* PfO-1 (score 514).

Therefore, pairwise sequence-based and functional comparisons of *P. citronellolis* E5 vs*. R. erythropolis* D4 and vs*. P. aeruginosa* PAO1 genomes were also carried out through RAST. As expected, the highest number of CDSs (644 genes) with more than 90% identity were shared between E5 and PAO1 genomes, which also showed 27% of shared functional gene categories. Instead, E5 vs. D4 genomes shared 17% of functional genes, and 302 genes of the E5 genome shared 50% identity compared to *R. erythropolis* D4.

**FIGURE 9:**
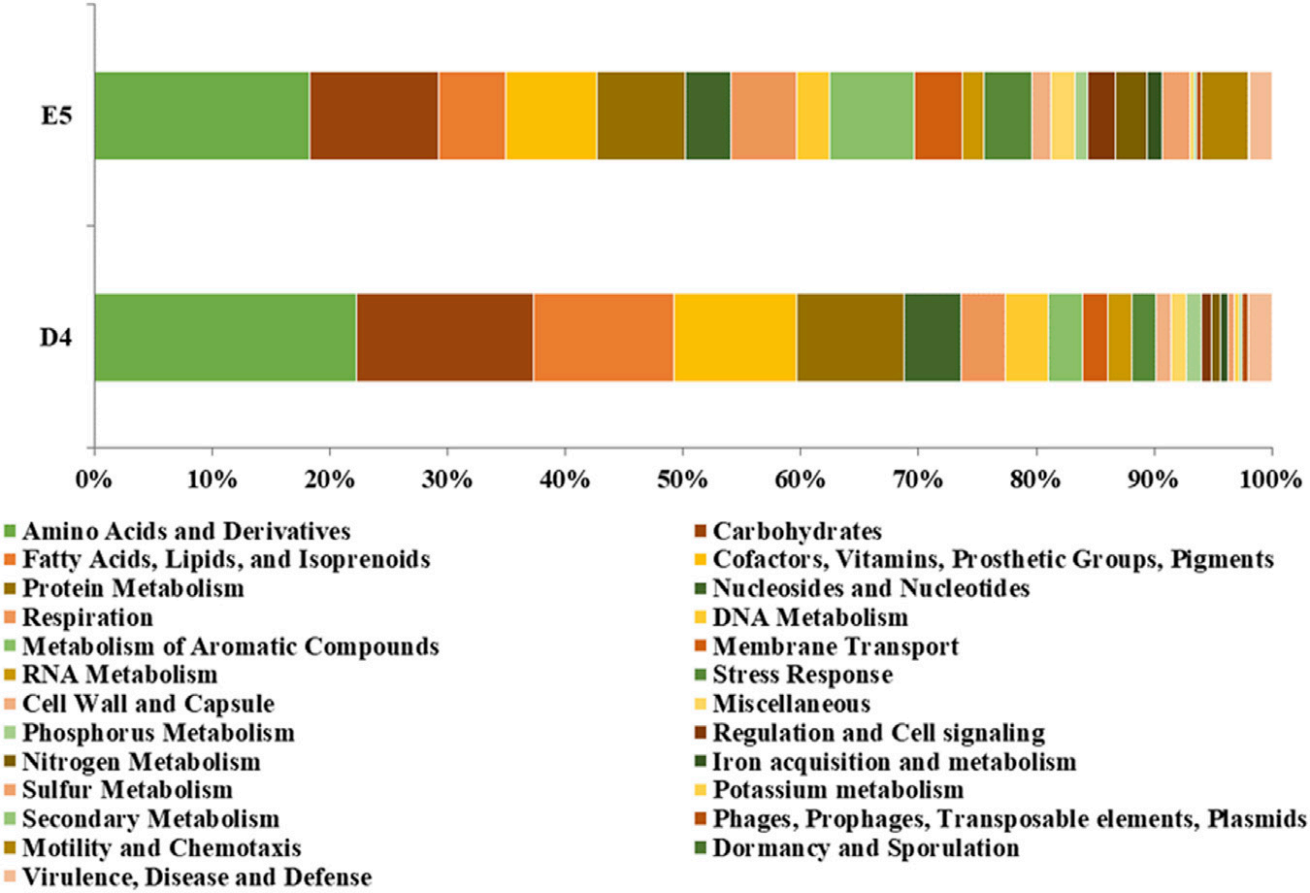
Percentage of CDSs in each RAST subsystem category for *P. citronellolis* E5 and *R*. *erythropolis* D4.

The RAST annotated CDSs of the *P. citronellolis* E5 genome over 386 subsystems shared in 25 categories (Overbeek et al., 2005) are 43%, among which amino acids and derivatives (518), carbohydrates (310), cofactors, vitamins, prosthetic groups or pigments (217), protein metabolism (214), and metabolism of aromatic compounds (203) are the most populated (Figure 9). A similar amount of CDS distribution in RAST subsystem categories is observed in the D4 genome, for which 37% of CDSs are categorized, except that fatty acids, lipids, and isoprenoids is the third-most-populated category.

### 3.7 Genetic trait prediction in *P. citronellolis* E5 and *R. erythropolis* D4 genomes for PE biodegradation

**FIGURE 10:**
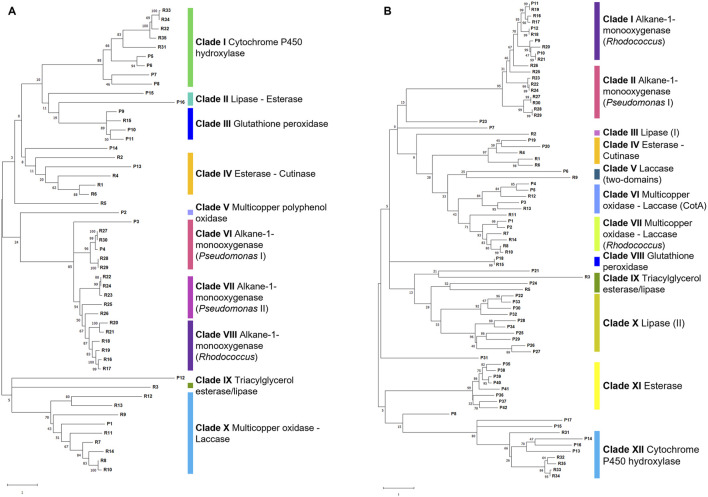
Cluster tree of selected gene products (named P) from *P. citronellolis* E5 **(A)** and *R. erythropolis* D4 **(B)** genomes against RAS (named R) (listed in Supplementary Table S2). Each clade is highlighted with a different color and named according to the RAS representing each clade. The clade number is in order from the top of each tree. The numbers on the branches represent the bootstrap values calculated for the ML method from the MEGA software with 50 bootstraps. Protein name abbreviations are reported in Supplementary Tables S2, S3 for *P. citronellolis* E5 and *R. erythropolis* D4, respectively.

The whole-genome comparative analysis of *P. citronellolis* E5 and *R. erythropolis* D4 showed distinctive traits. Two approaches to gene predictive mining were undertaken for *P. citronellolis* E5 and *R. erythropolis* D4 to predict the main functions involved in PE biodegradation. The first one involved a genome-based search focused on literature reports, knowledge about the main steps of the PE metabolism, and gene products retrieved from NCBI relying on annotation and aa sequence alignments. The annotation search extracted the following enzymatic classes: laccase-like multicopper oxidases, peroxidase, alkane monooxygenases, cytochrome P450 hydroxylases, lipases, and esterases. The second approach included the selection of 35 reference aa sequences (RASs) to frame the subsequent clusterization of gene products and to use as input against the E5 and D4 genomes (Supplementary Table S1; Supplementary Figure S2). The generated RAS tree is divided into ten clades (Supplementary Figure S2): clades I, II, III, IV, and V include (alkane) monooxygenases from different genera (*Rhodococcus*, *Acinetobacter*, *Pseudomonas*), clades VI and VII include respectively lipases and esterases, and clades VIII, IX, and X comprise cytochrome P450 hydroxylases, peroxidases, and multicopper oxidases-laccases, respectively. Thus, the selected 35 RASs were compared against the E5 and D4 strain genomes. The retrieved aa sequences from the annotation search (first approach) were also compared against 35 RASs. Overall, the aa sequences potentially involved in PE biodegradation according to the proposed metabolic pathway showing the highest identity percentage were selected (Supplementary Tables S2, S3). For each strain, the resulting gene products (16 for E5 and 42 for D4) deriving from these two approaches were aligned, and a cluster tree was developed showing respectively ten and twelve clades according to the RAS (aa sequence named R) clusterization (Figure 10A, B). The first metabolic step envisages laccase-like multicopper oxidases (Gravouil et al., 2017; Zampolli et al., 2021), peroxidases (Rong et al., 2024), esterases (Tao et al., 2023), or lipases (Kim et al., 2024) acting in the extracellular environment for the preliminary oxidation of PE. For this reason, only the gene products predicted to be potentially extracellular-secreted were chosen. *P. citronellolis* E5 evidenced two distinct multicopper oxidases (clades V and X Figure 10A), but only P1 (annotated as multicopper oxidase) showed similarity with COG2132 (multicopper oxidase with three cupredoxin domains), while P2 grouped alone (multicopper polyphenol oxidase). On the other hand, *R. erythropolis* D4 possesses five gene products annotated as multicopper oxidases that were distributed into clades VI and VII (Figure 10B). In addition, P6 was annotated as HP clustered with R9, suggesting that it could be an additional multicopper oxidase (clade V Figure 10B).

The number of glutathione peroxidases varies for the two strains: *P. citronellolis* E5 and *R. erythropolis* D4 showed three glutathione peroxidases (COG0386, clade III Figure 10A) and one glutathione peroxidase (clade VIII Figure 10B), respectively.

Among 45 esterases and 11 lipases annotated in the E5 strain genome and 69 esterases and 29 lipases in the D4 genome, only 5 and 24 gene products were retrieved from the whole genome of, respectively, the E5 and D4 strains based on the two approaches. Only P13 of E5 and P19 and P20 of D4 share the highest identity to esterases R1, R4, and R6 (clade IV Figures 10A, B; Supplementary Table S1), and P24 of D4 is similar to triacylglycerol esterases/lipases R3 and R5 (clade IX Figure 10B; Supplementary Table S1). Regarding lipases, only clade II for the E5 tree (Figure 10A) and clade X in the D4 tree (Figure 10B) include potential lipases, even if they are clustered without RASs.

The subsequent transport of oxidized products is mainly carried out by membrane transporters (Gravouil et al., 2017; Zampolli et al., 2021). This route is feasible in the selected strains because *P. citronellolis* E5 and *R. erythropolis* D4 account for around 390 and 375 transport proteins (in the RAST server), respectively (Figure 9). Moreover, because ABC transporters have been reported to be involved in PE oxidized product transportation, the two genomes were scanned for these potential gene products, recovering 302 and 277 potential ABC transporters for the E5 and D4 strains, respectively.

The oxygenated or lower molecular weight products are then intracellularly oxidated by oxygenases such as alkane monooxygenases/hydroxylases and cytochrome P450 hydroxylases (Gravouil et al., 2017; Zampolli et al., 2021; Tao et al., 2023; Mishra et al., 2024; Rong et al., 2024). Their search showed two and four alkane monooxygenases and four and five cytochrome P450 hydroxylases, respectively, in the E5 and D4 genomes. Consistently, in E5 clusterization, only P4, an alkane monooxygenase, belongs to clade VI (alkane-1-monooxygenase - *Pseudomonas* I), and all four cytochrome P450 hydroxylases (P5-P8) to clade I (Figure 10A). In D4 clusterization, all four alkane monooxygenases (P9-P12) belong to clade I (alkane-1-monooxygenase - *Rhodococcus*), and all five cytochrome P450 hydroxylases belong to clade XII (cytochrome P450 hydroxylases) (Figure 10B).

Gene products involved in the central metabolic pathway of β-oxidation are also known to be involved in the last metabolic steps (Tao et al., 2023; Mishra et al., 2024). Both strains possess approximately 40% of the gene products required for the β-oxidation metabolism in the selected KEGG map (Supplementary Figure S6). Each functional step is associated with diverse EC numbers, including long-chain-fatty-acid-CoA ligase, oxidoreductases, enoyl-CoA hydratase, 3-hydroxyacyl-CoA dehydrogenase, acetyl-CoA acyltransferase, 3-hydroxybutyryl-CoA epimerase, alcohol dehydrogenase, aldehyde dehydrogenase, and alkane 1-monooxygenase (EC: 6.2.1.3, 1.3.99.2, 4.2.1.17, 1.1.1.35, 2.3.1.16, 2.3.1.9, 5.1.2.3, 1.1.1.1, 1.2.1.3, and 1.14.15.3). A few differences between the two strains are evidenced in the β-oxidation metabolic pathway, which comprises dodecenoyl-CoA isomerase and rubredoxin reductase (EC numbers 5.3.3.8, 1.18.1.1) for E5 and fatty acyl-CoA oxidase (EC 1.3.3.6) for D4 (Supplementary Figure S6).

## 4 Discussion

The degradation of PE employing microorganisms is extensively investigated because this polyolefin is one of the most produced and the most recalcitrant. The identification of new bacterial isolates and investigation of their biodegradative metabolism enriches the knowledge of this current challenge.

The present work aims to characterize the PE biodegradative potential and identify plausible genomic and genetic traits of novel bacterial isolates from a solid organic waste rich in plastic debris derived from a waste treatment plant. Indeed, these kinds of samples are rich in microbial biodiversity, and the presence of diverse plastics can guarantee the identification of bacteria with metabolic capabilities (Devi et al., 2023; Mishra et al., 2024; Zampolli et al., 2024). Among other isolates, *P. citronellolis* E5 was isolated from organic waste rich in plastic and selected for its interesting properties toward PE oxidation. To the best of our knowledge, the E5 strain is the first isolate belonging to *P. citronellolis* species whose biodegradative profile toward PE was characterized by both metabolic and biodegradative assays and from the genetic and genomic point of view. The other two reports regarding *P. citronellolis* strains able to biodegrade polyethylene characterized their potential only at the metabolic and phenotypic levels (Pramila et al., 2012; Bhatia et al., 2014). It is interesting to notice that these other *P. citronellolis* strains were also isolated from municipal waste.

Among bacteria with intriguing plastic-degrading capacities, bacteria of the *Rhodococcus* genus are well known for their degrading features against emerging contaminants, including plastic (Alvarez, 2019; Gravouil et al., 2017). *R*. *erythropolis* species present genetic traits that lead to speculation that they could efficiently contribute to PE biodegradation or reduction (Zampolli et al., 2022). Thus, *R. erythropolis* D4 (Zampolli et al., 2024) was selected as a comparative strain to study the biodegradation of PE in an eco-friendly process with a low toxic by-product generation.

Here, we reported a framework to study and evaluate microbial PE potential based on phenotypic observation and bioinformatic predictions viable for both Gram-negative and Gram-positive bacteria.

Among other isolates, *P. citronellolis* E5 and *R*. *erythropolis* D4 were assayed and proved capable of growing on untreated commercial powder PE as the sole carbon and energy source for up to 60 days, showing a high number of bacterial cells already at 7 days. Often, to study the biodegradative potential of polyolefins, the chosen polymer is subjected to pretreatment to facilitate bacterial attack. Only a few studies focused on untreated PE to evaluate the full potential of microbial cells (Devi et al., 2023; Tao et al., 2023). Biodegradative studies focusing on PE reduction or elimination showed that untreated LDPE could be degraded by different strains of *Pseudomonas* (Kyaw et al., 2012; Yoon et al., 2012; Montazer et al., 2020). In this case, we mimicked the condition of PE microplastic because the biodegradative growth assays were carried out with PE powder.

The secreted and intracellular laccase activity were tested to support the isolate screening for PE biodegradation. Indeed, literature reports bacteria able to activate laccase-like enzymes to perform the first oxidation of PE (Santo et al., 2013; Gravouil et al., 2017; Zampolli et al., 2021; Tao et al., 2023). In this case, all the selected bacteria were able to secrete active laccases during growth on PE, ensuring its initial attack (E5 strain showed an activity equal to ((2±1) *10^−3^ U mg^−1^, D4 strain of around (3±1) *10^−3^ U mg^−1^) (Zampolli et al., 2021; Tao et al., 2023).

In line with this data, GC-MS analysis provides direct evidence of PE depolymerization derived from the profile of their growth and the corresponding metabolic products from PE oxidation. For both strains, an oscillatory pattern of PE products was observed because every generated oxidized product (medium- and long-chain alkanes and carboxylic acids) was subsequently degraded at the next sampling time. In this regard, the preliminary growth assays on representative metabolic intermediates of PE biodegradation, including alkanes, ketones, and carboxylic acids, supported these biodegradative trends for both strains. A few differences in terms of metabolic products and detection time were evidenced by the two strains, and great differences were detected between the control (no inoculum) and the unprocessed commercial PE powder (t0). It is worth mentioning that the initial PE analyses suggested a partial oxidation of the polymer due to its production process, as already reported by Zampolli et al. (2023). A few products were similar to the one detected by Rong et al. (2024) during the degradation of a branched LDPE by *Nitratireductor* sp. Z-1 and *Gordonia* sp. Z-2 (alkanes and fatty acids). Probably, this is due to a double extraction of the supernatant of each culture using ethyl acetate and DCM; thus, they also observed short-chain alkanes, alkanols, and esters. The present work used DCM extraction. The two strains could oxidize not only the polymer chains but also all the oxidized and non-oxygenated products deriving from the PE attack. This behavior is not surprising for D4 strains because, in a previous study, the D4 strain was demonstrated to be able to utilize diverse carboxylic acids from 9 to 20 carbons in the aliphatic chain (Zampolli et al., 2024). Moreover, the *R*. *erythropolis* species is known to possess degradative abilities toward aliphatic hydrocarbons (de Carvalho and da Fonseca, 2005; Zampolli et al., 2019), and some *P*. *citronellolis* bacteria were reported to be capable of biodegrading alkanes (Koutinas et al., 2021).

In order to correlate the meaningful phenotypic data on PE with the main genomic and genetic features, the genome of *P. citronellolis* E5 was completely sequenced, analyzed, and compared with the genome of *R*. *erythropolis* D4 (Zampolli et al., 2024). The size and GC percentage of the E5 genome are in line with that of the other strains of *P. citronellolis* species (NCBI taxid 53408) and highly divergent from that of *R*. *erythropolis* D4, with an ANI below 80% (Zampolli et al., 2023).

Whole-genome alignments, annotation search, and gene clustering against reference gene products (RAS) were exploited based on literature reports and knowledge about the main steps of the PE metabolism: (i) initial attack and oxidation of PE polymer, (ii) internalization transport of oxidized products, and (iii) subsequent intracellular oxidation of oxygenated or lower molecular weight products. In relation to the main enzymatic classes involved in the first step, the classes considered included laccase-like multicopper oxidases, peroxidase, lipases, and esterases (Gravouil et al., 2017; Montazer et al., 2020; Zampolli et al., 2021; Tao et al., 2023; Mishra et al., 2024; Rong et al., 2024; Kim et al., 2024). *P*. *citronellolis* E5 exhibited a higher number of glutathione peroxidases than *R*. *erythropolis* D4, and the opposite proportion was observed for multicopper oxidases. As reported by Rong et al. (2024), glutathione peroxidases can initiate the cleavage process of C-C bonds and C-H bonds in the polymer, leading to physical PE destruction. This could be likely the case of the E5 strain that produced shorter aliphatic products than the D4 strain over time, which, in conversion, produced more medium- and long-chain alkanes potentially generated from the multicopper oxidase initial attack. In line with this, it is not possible to exclude the involvement of other oxidases for PE initial oxidation for the two strains.

A few esterases were detected in the genomes, and no lipases clustered with reference sequences for both strains. These esterases/lipases can participate in the first attack in the case of PE products with ester bonds in the oxidized backbone (Kim et al., 2024).

The subsequent oxygenated or low molecular PE fragments require the presence of transportation gene products, especially ABC transporters, for the plausible entrance into the cells (Gravouil et al., 2017; Zampolli et al., 2021). Indeed, numerous genes encoding transporters were detected in both genomes.

The main enzymatic classes potentially involved in the last step comprise intracellular enzymes, and we considered alkane monooxygenases and cytochrome P450 hydroxylases (Gravouil et al., 2017; Zampolli et al., 2021; Tao et al., 2023; Mishra et al., 2024; Rong et al., 2024; Kim et al., 2024). Bacteria belonging to the *Pseudomonas* and *Rhodococcus* genera are well known to possess these oxidative systems to degrade alkanes (Zampolli et al., 2019), and the bacteria considered showed multiple gene products potentially related to PE oxidation. Indeed, Yoon et al. (2012) and Jeon and Kim (2015) demonstrated that *alkB* from *P. aeruginosa* E4 and the *alk* gene cluster of *P. aeruginosa* E7 actively degraded LDPE. In the case of the E4 strain, this occurred even in the absence of rubredoxins and rubredoxin reductase.

These investigations provided a set for each strain of genomic traits and gene products potentially involved in PE biodegradation for each of the PE biodegradative steps, strengthening the PE mineralization potential in both selected bacteria.

## 5 Conclusion

In the present work, novel bacterial isolates were characterized for PE biodegradative potential. A bacterium belonging to the *P. citronellolis* species was selected and characterized to develop a biological eco-friendly process to eliminate PE. In addition to growth and biodegradative comparative investigations with respect to the efficient plastic-degrading *R*. *erythropolis* D4, in-depth genomic and genetic determinant exploration was performed, providing a toolbox for both Gram-negative and Gram-positive bacteria useful for multiple biotechnological applications toward PE biodegradation. Moreover, these bacterial comparative analyses pave the way for combining multiple bacteria strains to cope with the prominent issue of polyolefin degradation.

## Data Availability

The datasets presented in this study can be found in online repositories. The name of the repository and accession number can be found in the article.
