# Supplementary figures and images for: Insights into polyethylene biodegradative fingerprint of Pseudomonas citronellolis E5 and Rhodococcus erythropolis D4 by phenotypic and genome-based comparative analyses
Source: Front Bioeng Biotechnol. 2024 Dec 12;12:1472309. doi: 10.3389/fbioe.2024.1472309 (PMC11669507; doi:10.3389/fbioe.2024.1472309)

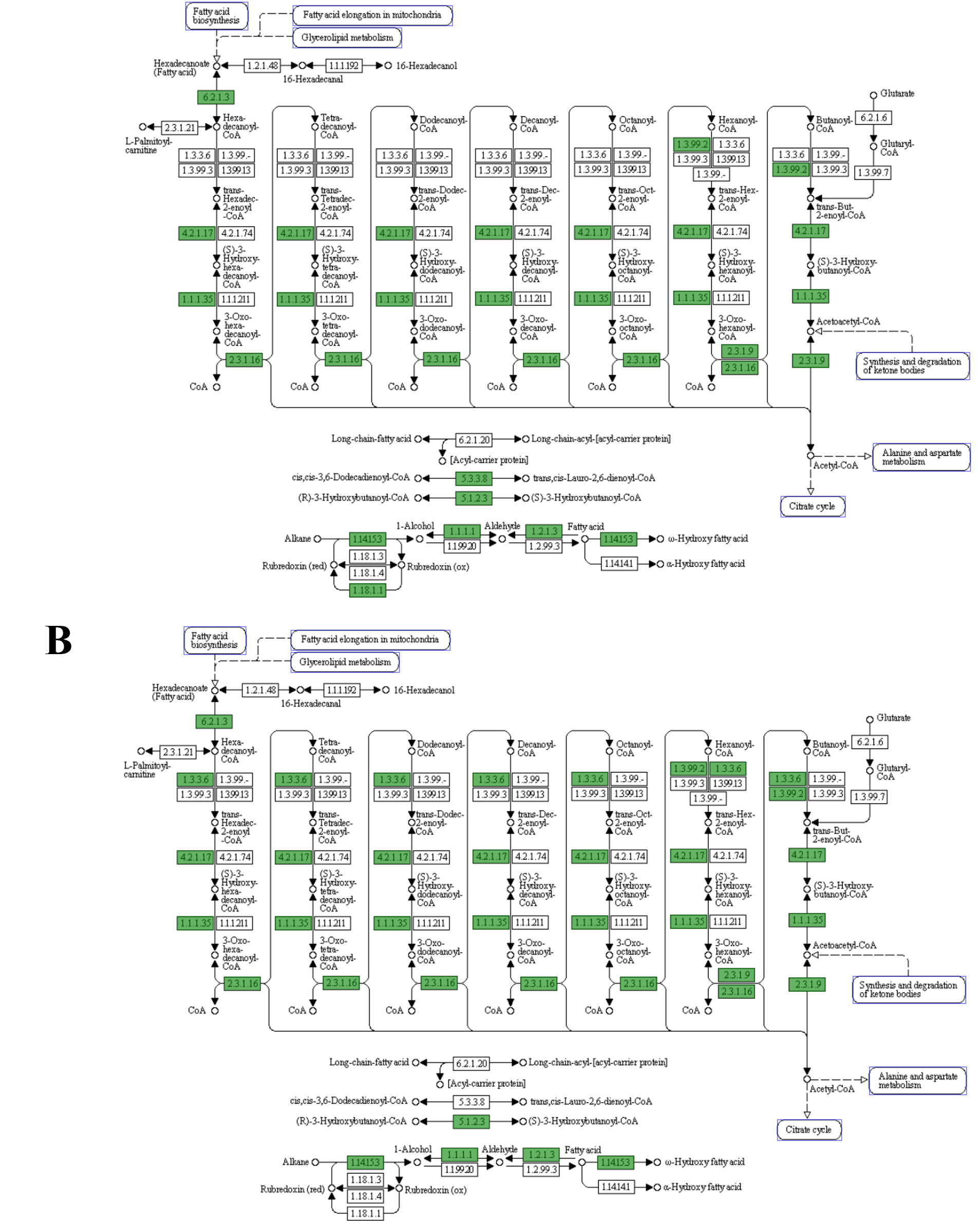

Supplement: Supplementary file 1 [file Image6.TIF]

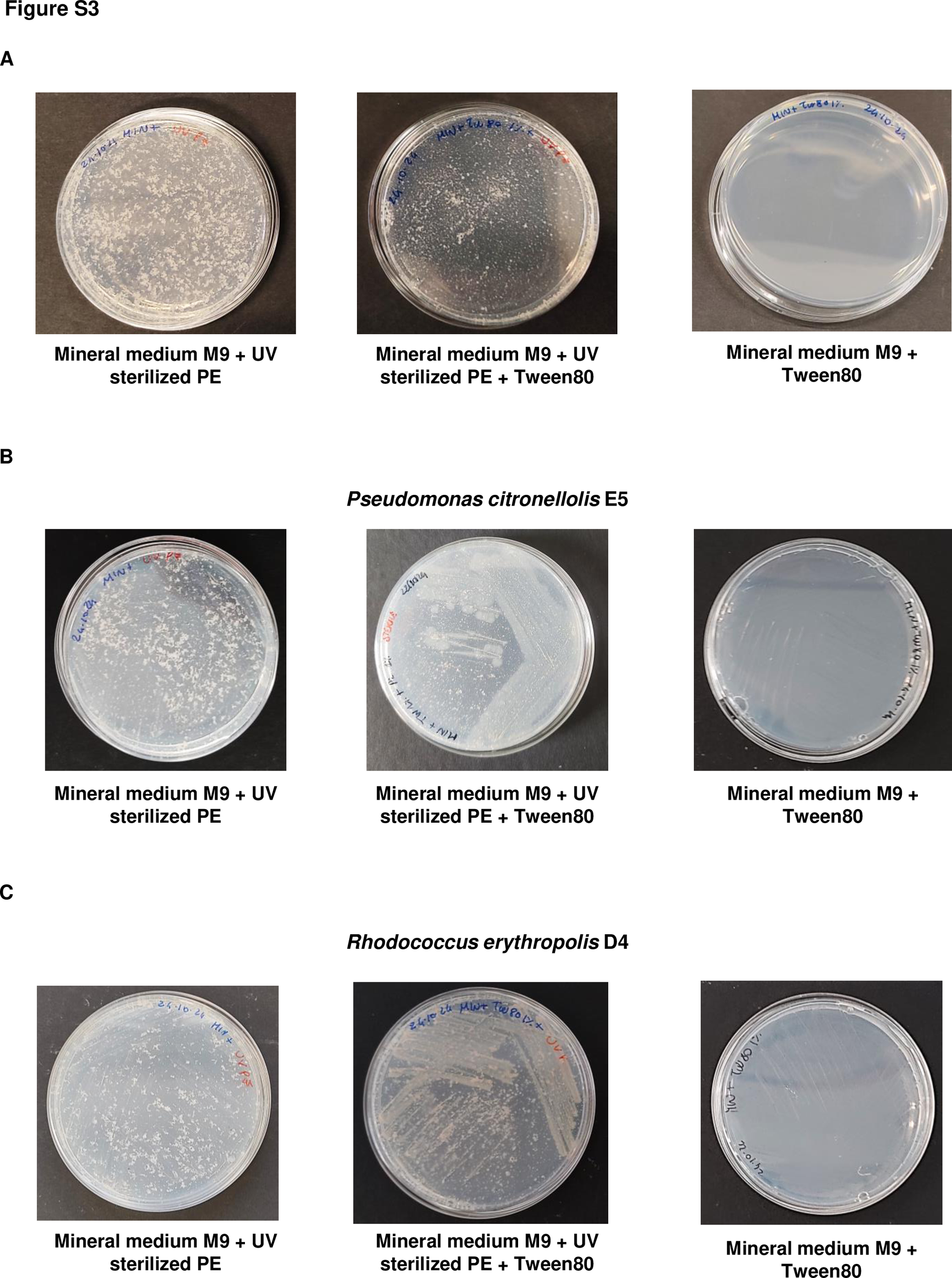

Supplement: Supplementary file 3 [file Image3.TIF]

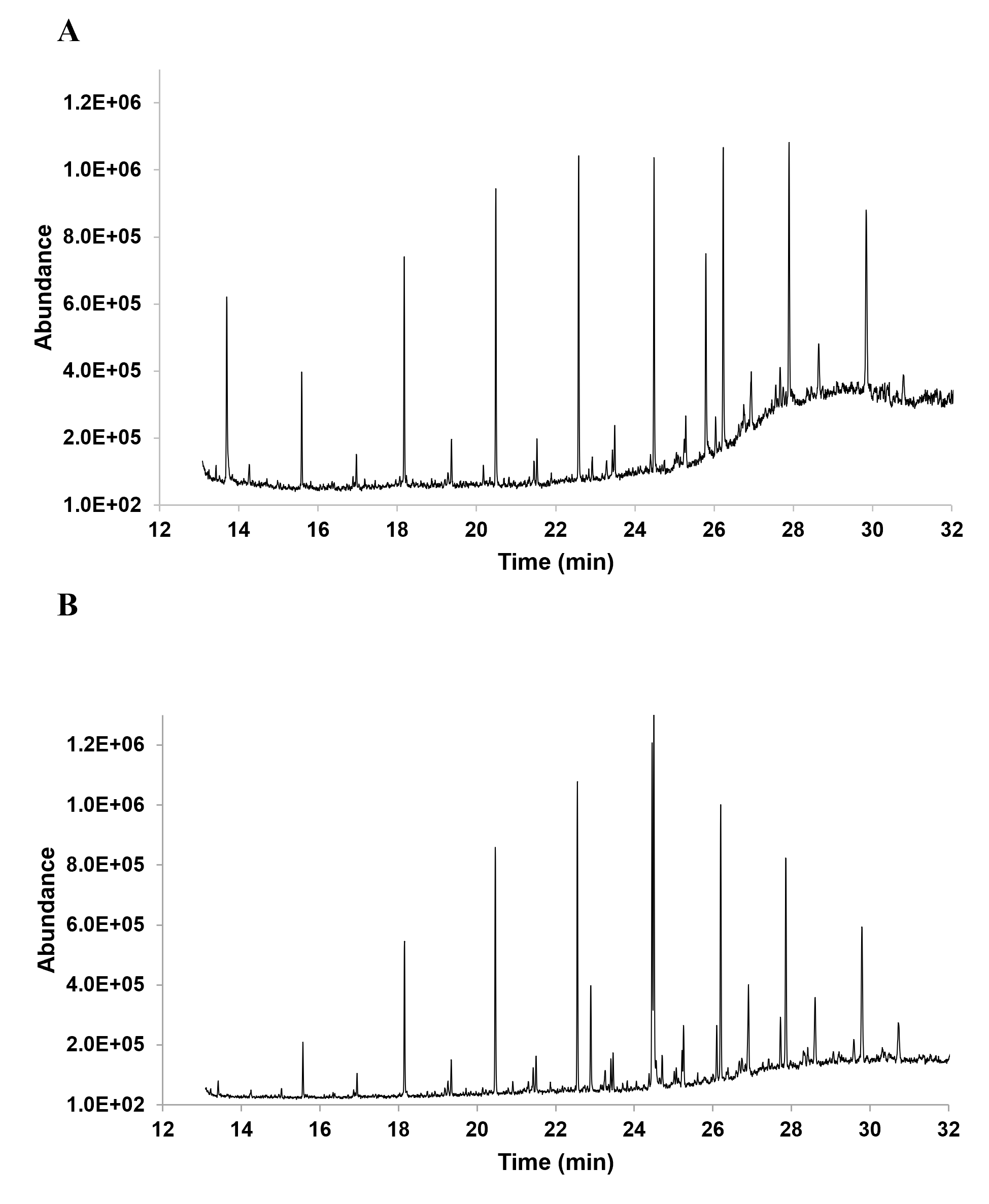

Supplement: Supplementary file 4 [file Image4.TIF]

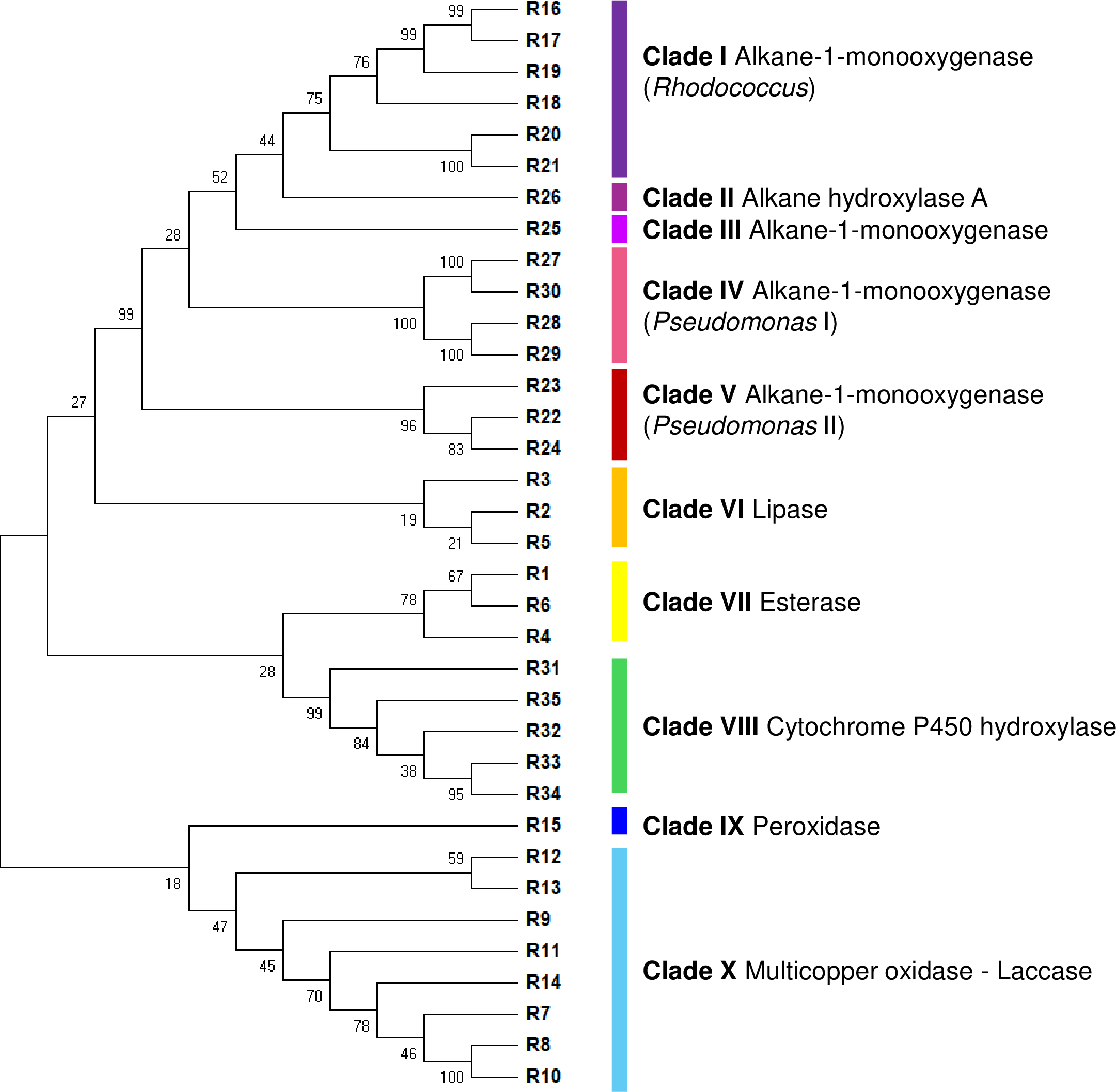

Supplement: Supplementary file 5 [file Image2.TIF]

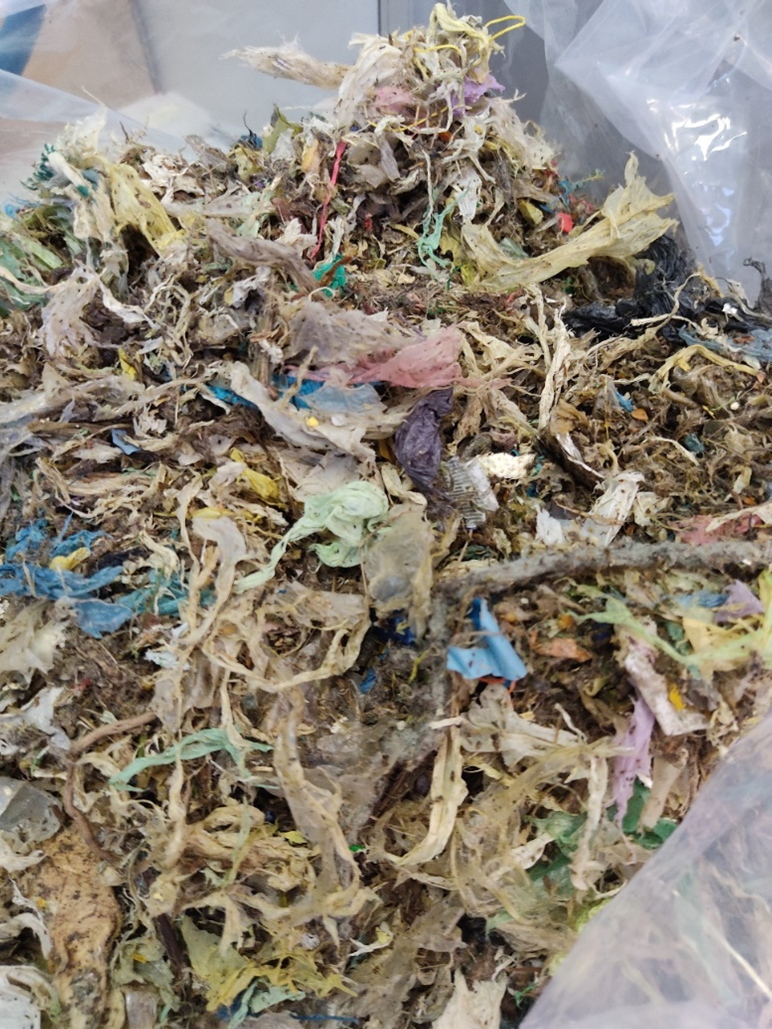

Supplement: Supplementary file 6 [file Image1.TIF]

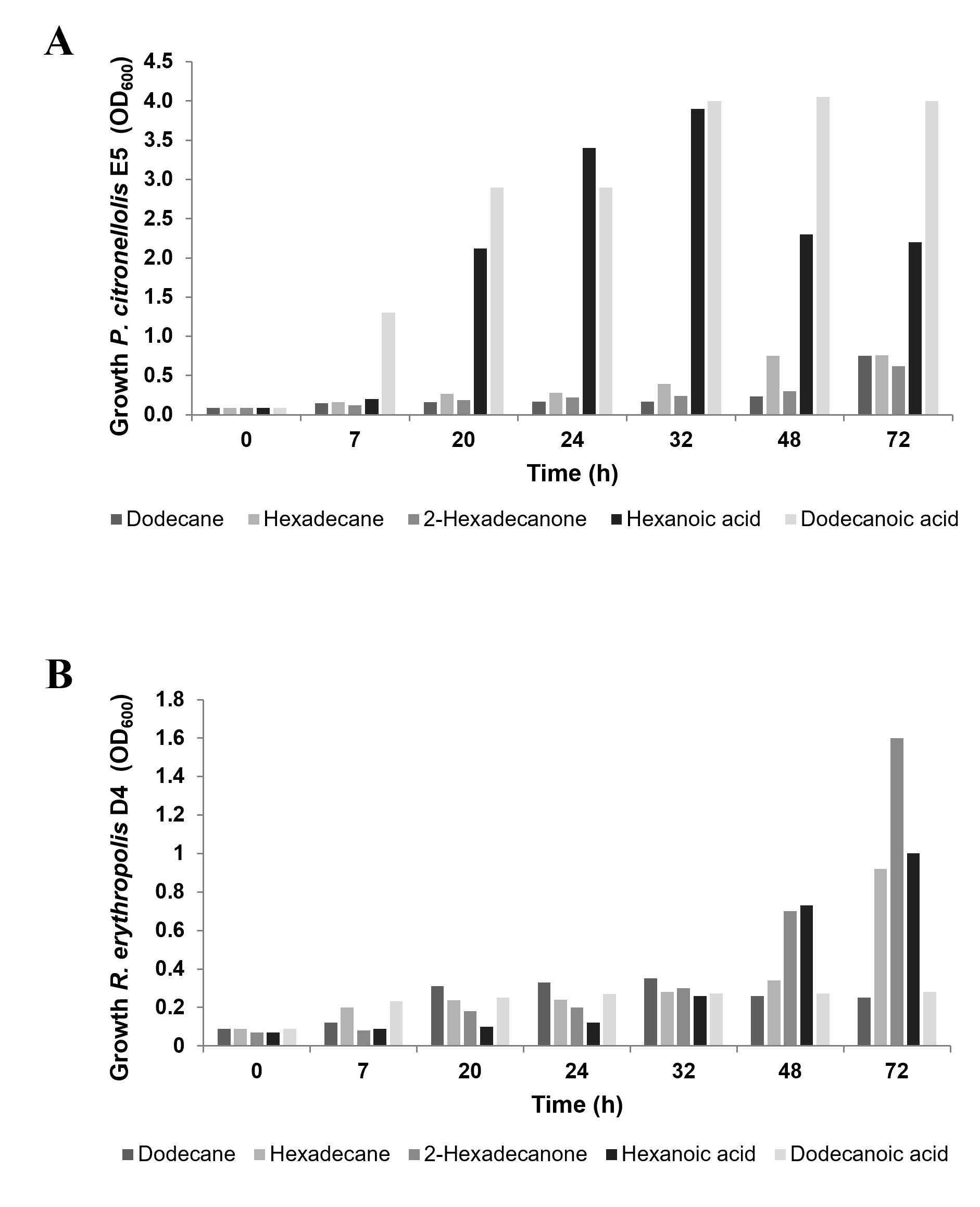

Supplement: Supplementary file 9 [file Image5.TIF]
