# Supplementary material for: Insights into polyethylene biodegradative fingerprint of Pseudomonas citronellolis E5 and Rhodococcus erythropolis D4 by phenotypic and genome-based comparative analyses
Source: Front Bioeng Biotechnol. 2024 Dec 12;12:1472309. doi: 10.3389/fbioe.2024.1472309 (PMC11669507; doi:10.3389/fbioe.2024.1472309)
Supplement: Supplementary file 2 [file Table1.DOCX]

**Table S1. List of reference amino acid sequences (RAS)** **from different microorganisms involved in PE metabolism deriving from a manual search of the literature and databases.**

| **Name used for the clusterization** | **NCBI accession ID** | **UniProt accession ID** | **Gene accession number^a^** | **Protein accession number^a^** | **Type** | **Source** | **References** |
| --- | --- | --- | --- | --- | --- | --- | --- |
| **Esterase** | | | | | | | |
| **R1** | BAI99230 | D4Q9N1 | AB445476 | BAI99230 | Esterase | *Thermobifida alba* | Hu et al. 2010 |
| **Lipase** | | | | | | | |
| **R2** | 4ZV7^b^ | P41365 | Z30645 | CAA83122 | Chain A, Lipase B | *Moesziomyces antarcticus* | Strzelczyk et al. 2015; Shi et al., 2020 |
| **R3** | 5AH0 | A0A0A0YMQ9 | KM377649 | AIX10936.1 | Triacylglycerol esterase/lipase EstA | *Pelosinus fermentans* DSM 17108 | Biundo et al. 2016 |
| **R4** | WP_004373894 | A0A8I0SEY2 | JADMNJ010000005 | MBF8162059 | Hypothetical protein | *Pseudomonas mendocina* | Li et al. 2022 |
| **R5** | WP_003239806 | A0A8I0SFQ4 | JADMNJ010000002 | MBF8160636 | Triacylglycerol lipase | *Pseudomonas mendocina* | Li et al. 2022 |
| **R6** | ADK73612 | E9KJL1 | GU592443 | ADK73612 | Cutinase A precursor | *Pseudomonas oleovorans* | Inglis et al. 2011 |
| **Multicopper oxidase - Laccase** | | | | | | | |
| **R7** | AII08809 | - | CP008947 | AII08809 | Multicopper oxidase | *Rhodococcus opacu*s R7 | Zampolli et al. 2021 |
| **R8** | AII11185 | - | CP008950 | AII11185 | Multicopper oxidase | *Rhodococcus opacu*s R7 | Zampolli et al. 2021 |
| **R9** | AII11221 | - | CP008950 | AII11221 | Multicopper oxidase | *Rhodococcus opacu*s R7 | Zampolli et al. 2021 |
| **R10** | AII09363 | - | CP008947 | AII09363 | Multicopper oxidase | *Rhodococcus opacu*s R7 | Zampolli et al. 2021 |
| **R11** | UVG67878 | - | OM891104 | UVG67878 | Laccase | *Psychrobacter* sp. | Zhang et al. 2022 |
| **R12** | WP_003243170 | P07788 | CP053102 | - | Outer spore coat copper-dependent laccase (CotA) | *Bacillus subtilis* | Yao et al. 2022 |
| **R13** | AAC73234 | P36649 | U00096 | AAC73234 | Multicopper oxidase CueO | *Escherichia coli* K12 | Blattner et al. 1997 |
| **R14** | - | - | - | - | Multicopper oxidase - laccase | *Rhodococcus ruber* C208 | Santo et al. 2013 |
| **Peroxidase** | | | | | | | |
| **R15** | WP_003945816 | - | CP078080 | WP_003945816 | GPx Glutathione Peroxidase | *Rhodococcus* sp. C-2 | Rong et al. 2024 |
| **Oxygenase** | | | | | | | |
| **R16** | AIA09965 | - | KJ573524 | AIA09965 | Alkane-1 monooxygenase EC 1.14.15.3 | *Rhodococcus opacu*s R7 | Zampolli et al. 2014; Zampolli et al. 2021 |
| **R17** | ADR72654 | E5G6V9 | HM771646 | ADR72654 | Alkane-1-monooxygenase | *Rhodococcus aetherivorans* BCP1 | Cappelletti et al. 2011; Cappelletti et al. 2010 |
| **R18** | CAB51053 | Q9XAU0 | AJ009586 | CAB51053 | Alkane-1-monooxygenase | *Rhodococcus erythropolis* Q15 | Smits et al. 1999 |
| **R19** | CAC37038 | Q9AE68 | AJ297269 | CAC37038 | Alkane-1-monooxygenase | *Rhodococcus erythropolis* Q15 | Smits et al. 1999 |
| **R20** | CAC40953 | Q93JS2 | AJ301876 | CAC40953 | Alkane-1-monooxygenase | *Rhodococcus erythropolis* Q15 | Smits et al. 1999 |
| **R21** | CAC40954 | Q93JS1 | AJ301877 | CAC40954 | Alkane-1-monooxygenase | *Rhodococcus erythropolis* Q15 | Smits et al. 1999 |
| **R22** | CAB54050 | P12691 | AJ245436 | CAB54050 | Alkane-1-monooxygenase | *Pseudomonas putida* TF4-1L | Kok et al. 1989 |
| **R23** | CAB51047 | Q9WWW6 | AJ233397 | CAB51047 | Alkane-1-monooxygenase | *Pseudomonas putida* P1 | van Beilen et al. 2001 |
| **R24** | AHY94988 | A0A024A3Z2 | KJ489379 | AHY94988 | Alkane-1-monooxygenase | *Pseudomonas citronellolis* UAM-Ps1 | Bravo et al. 2015 |
| **R25** | BAB33284 | Q9AQK2 | AB049410 | BAB33284 | Alkane hydroxylase A | *Acinetobacter* sp. M-1 | Tani et al. 2001 |
| **R26** | CAB51045 | Q9XAV1 | AJ009579 | CAB51045 | Alkane 1-monooxygenase | *Pseudomonas protegens* CHA0 | Smits et al. 1999 |
| **R27** | ANI09868 | - | CP015877 | ANI09868 | Alkane 1-monooxygenase | *Pseudomonas aeruginosa* SJTD-1 | Liu et al. 2012 |
| **R28** | ANI10903 | - | CP015877 | ANI10903 | Alkane 1-monooxygenase | *Pseudomonas aeruginosa* SJTD-1 | Liu et al. 2012 |
| **R29** | NP_251264 | Q9I0R2 | PA2574 | NP_251264 | Alkane 1-monooxygenase | *Pseudomonas aeruginosa* PAO1 | Smits et al. 2003 |
| **R30** | NP_250216 | Q6H941 | PA1525 | NP_250216 | Alkane 1-monooxygenase | *Pseudomonas aeruginosa* PAO1 | Smits et al. 2003 |
| **R31** | AII08421 | - | CP008947 | AII08421 | Cytochrome P450 | *Rhodococcus opacus* R7 | Zampolli et al. 2021 |
| **R32** | CAC37902 | Q93SX5 | AJ311718 | CAC37902 | Alkane monooxygenase | *Acinetobacter* sp. EB104 | Maier et al. 2001 |
| **R33** | CAH59967 | Q5K134 | AJ844908.1 | CAH59967 | Cytochrome P450 alkane hydroxylase | *Alcanivorax borkumensis* | van Beilen et al. 2006 |
| **R34** | CAH59968 | Q5K133 | AJ844909.1 | CAH59968 | Cytochrome P450 alkane hydroxylase | *Alcanivorax borkumensis* | van Beilen et al. 2006 |
| **R35** | KZL95198 | UPI00079C11C7 | XX05_11415 | KZL95198 | Cytochrome | *Tsukamurella tyrosinosolvens* PS2 | Romanova et al. 2022 |

^a^ NCBI accession number, the ID derives from EMBL, GenBank, or DDBJ databases.

^b^ 4ZV7, the accession number derives from PDB database.
