# Supplementary material for: Insights into polyethylene biodegradative fingerprint of Pseudomonas citronellolis E5 and Rhodococcus erythropolis D4 by phenotypic and genome-based comparative analyses
Source: Front Bioeng Biotechnol. 2024 Dec 12;12:1472309. doi: 10.3389/fbioe.2024.1472309 (PMC11669507; doi:10.3389/fbioe.2024.1472309)
Supplement: Supplementary file 7 [file Table2.DOCX]

**Table S2. Gene products of *P. citronellolis* E5 used for clustering analysis with RAS proteins denominated according to P-number, specifying the putative functions and ID from RAST.**

| **Name used for the clusterization** | **Functions from RAST** | **RAST ID** |
| --- | --- | --- |
| **P1** | Multicopper Oxidase | 3538 |
| **P2** | Multicopper polyphenol oxidase | 5112 |
| **P3** | Alkane-1 monooxygenase | 960 |
| **P4** | Alkane-1 monooxygenase | 4688 |
| **P5** | Cytochrome P450 hydroxylase | 1332 |
| **P6** | Cytochrome P450 hydroxylase | 1369 |
| **P7** | Putative cytochrome P450 hydroxylase | 3317 |
| **P8** | Putative cytochrome P450 hydroxylase | 4253 |
| **P9** | Glutathione peroxidase | 3843 |
| **P10** | Glutathione peroxidase | 1553 |
| **P11** | Glutathione peroxidase | 1321 |
| **P12** | Hypothetical protein | 2133 |
| **P13** | Probable lipoprotein signal peptide | 4640 |
| **P14** | Hypothetical protein (HP) | 376 |
| **P15** | Esterase / Lipase | 1583 |
| **P16** | Bifunctional outer membrane translocase / extracellular lipase, PlpD | 2319 |
