# Supplementary material for: Insights into polyethylene biodegradative fingerprint of Pseudomonas citronellolis E5 and Rhodococcus erythropolis D4 by phenotypic and genome-based comparative analyses
Source: Front Bioeng Biotechnol. 2024 Dec 12;12:1472309. doi: 10.3389/fbioe.2024.1472309 (PMC11669507; doi:10.3389/fbioe.2024.1472309)
Supplement: Supplementary file 8 [file Table3.DOCX]

**Table S3. Gene products of *R. erythropolis* D4 used for clustering analysis with RAS proteins denominated according to P-number, specifying the putative functions and ID from RAST.**

| **Name used for the clusterization** | **Function from RAST** | **RAST ID** |
| --- | --- | --- |
| **P1** | Multicopper oxidase | 609 |
| **P2** | Multicopper oxidase | 617 |
| **P3** | Multicopper oxidase | 3736 |
| **P4** | Multicopper oxidase | 5642 |
| **P5** | Multicopper oxidase | 5687 |
| **P6** | HP^a^ | 2722 |
| **P7** | HP | 4709 |
| **P8** | HP | 4729 |
| **P9** | Alkane-1 monooxygenase | 522 |
| **P10** | Alkane-1 monooxygenase | 3631 |
| **P11** | Alkane-1 monooxygenase | 3823 |
| **P12** | Alkane-1 monooxygenase | 5295 |
| **P13** | Putative cytochrome P450 hydroxylase | 1026 |
| **P14** | Putative cytochrome P450 hydroxylase | 2470 |
| **P15** | Cytochrome P450 | 2752 |
| **P16** | Cytochrome P450 | 4966 |
| **P17** | Putative cytochrome P450 | 5219 |
| **P18** | Glutathione peroxidase | 5068 |
| **P19** | HP | 152 |
| **P20** | HP | 4099 |
| **P21** | HP | 3488 |
| **P22** | Lipase LipV | 5425 |
| **P23** | Uncharacterized protein SCO0503 | 5271 |
| **P24** | Epoxide hydrolase (EC 3.3.2.9) | 2088 |
| **P25** | Lipase 1 (EC 3.1.1.3) | 240 |
| **P26** | Putative lipase | 449 |
| **P27** | Lipase 1 (EC 3.1.1.3) | 1025 |
| **P28** | Triacylglycerol lipase precursor (EC 3.1.1.3) | 2688 |
| **P29** | Probable triacylglycerol lipase (EC:3.1.1.3) | 2996 |
| **P30** | Putative lipase | 3109 |
| **P31** | Lipase, class 2 | 3749 |
| **P32** | Lipase LipV | 4237 |
| **P33** | Secreted lipase | 5426 |
| **P34** | Triacylglycerol lipase precursor (EC 3.1.1.3) | 6090 |
| **P35** | Putative esterase | 1831 |
| **P36** | Putative esterase | 1832 |
| **P37** | Putative esterase | 1974 |
| **P38** | Putative esterase | 2741 |
| **P39** | Putative esterase | 4541 |
| **P40** | Putative esterase | 4542 |
| **P41** | Putative esterase | 4617 |
| **P42** | Putative esterase | 5774 |

^a^ HP, Hypothetical Protein.
